# A sustainable house design to improve child health in rural Africa: a cluster-randomized controlled trial

**DOI:** 10.1038/s41591-026-04367-w

**Published:** 2026-04-21

**Authors:** Salum Mshamu, Mavuto Mukaka, Casiana Sanga, Stanslous Kambanga, Aneth Deogratias Rutaihwa, Thomas Chevalier Bøjstrup, Judith Meta, Arnold Mmbando, Christopher Pell, Bipin Adhikari, Ally Ibrahim Olotu, Ntuli Kapologwe, Nicholas J. White, Arjen M. Dondorp, Nicholas P. J. Day, Mallika Imwong, Jacqueline Deen, Steven W. Lindsay, Jakob Brandtberg Knudsen, Lorenz von Seidlein

**Affiliations:** 1 CSK Research Solutions, Mtwara, Tanzania; 2 Centre for Tropical Medicine and Global Health, Nuffield Department of Clinical Medicine, University of Oxford, Oxford, UK; 3 Mahidol−Oxford Tropical Medicine Research Unit (MORU), Centre for Tropical Medicine and Global Health, Bangkok, Thailand; 4 Royal Danish Academy – Architecture, Design, Conservation, Copenhagen, Denmark; 5 Private consultant, Ifakara, Tanzania; 6 Ifakara Health Institute, Ifakara, Tanzania; 7 Department of Biosciences, Durham University, Durham, UK; 8 Amsterdam Institute for Global Health and Development, Amsterdam, The Netherlands; 9 Amsterdam UMC, location University of Amsterdam, Department of Global Health Amsterdam, Amsterdam, The Netherlands; 10 Amsterdam Public Health Research Institute, Netherlands University of Amsterdam, Amsterdam, The Netherlands; 11 East, Central and Southern Africa Health Community (ECSA – HC), Arusha, Tanzania; 12 University of Philippines, Manila, Philippines

**Keywords:** Epidemiology, Epidemiology

## Abstract

Malaria, diarrhea and acute respiratory infections (ARIs) are the major causes of mortality in young children in sub-Saharan Africa. Here we provide support for the hypothesis that children can be protected from these diseases by improvements in house design. We designed a novel double-story house, called a Star Home, to provide an insect-proof, cleaner, cooler and smoke-free environment, with a reliable supply of water and sanitation. We conducted a cluster-randomized controlled trial where households with children under 13 years of age were randomly allocated (1:4.7) to living in 110 Star Homes or in 513 traditional mud and thatched-roofed houses. The primary outcome of childhood malaria incidence was met: after 3 years, children living in Star Homes had 44% less malaria (incidence rate ratio (IRR): 0.56 (95% confidence interval (CI): 0.43−0.72), *P* < 0.0001) compared to children living in traditional homes. Children in Star Homes had 30% less diarrhea (IRR: 0.70 (95% CI: 0.53−0.91), *P* = 0.0070) and 18% less ARIs (IRR: 0.82 (95% CI: 0.73−0.93), *P* = 0.0010) than children living in traditional homes. Children under 5 years of age living in Star Homes were also taller for their age than those living in traditional homes. Our house design is intended to inspire those working in the building sector and with local communities to develop innovative designs for healthier homes. Major improvements in rural house design have the potential to make a substantial public health impact across hot, humid regions of Africa. ClinicalTrials.gov: NCT04529434.

## Abstract (web summary)

A cluster-randomized trial in rural Tanzania showed that a sustainable housing design reduced incidences of malaria, diarrhea, acute respiratory infections and stunting in children under 5 years of age compared to traditional homes.

## Main

Malaria, diarrheal diseases and ARIs are three major causes of mortality in young children in sub-Saharan Africa^1,2^. Malaria accounts annually for an estimated 610,000 lives lost—an incidence more than three times higher than the global target^3^. In 2024, 282 million malaria cases were reported worldwide, nine million more than in 2023, with 94% of cases and 95% of deaths occurring in Africa. Diarrhea is responsible for approximately 1.8 million childhood deaths in low- and middle-income countries, of which approximately 375,000 occur in sub-Saharan Africa^4^. ARIs contribute up to one-third of under-5 mortality—approximately 350,000 deaths in sub-Saharan Africa^5^.

The transmission of these three preventable childhood killer diseases is closely associated with the home environment, where children spend most of their time^6,7^. Homes with suboptimal ventilation and infrequent air turnover can facilitate the transmission of airborne infections, particularly when an infected person coughs or sneezes in shared living spaces^8^. Overcrowding further increases the risk because close and prolonged contact among family members makes it easier for pathogens to spread^9,10^. Poor sanitation and insufficient access to clean water can create conditions that support the transmission of pathogens^11^. When households lack proper toilets, waste disposal systems or handwashing facilities, surfaces, food and water become contaminated. Poorly constructed homes attract and allow entry of mosquitoes, flies and rodents, which can spread infections^6,12^. Inadequate waste management and poor drainage around the home can create breeding sites for disease vectors, further increasing the risk of disease transmission. House designs that do not separate cooking, sleeping and sanitation areas can expose children to indoor air pollution, contaminated surfaces and infectious droplets^13^. Young children, who often play on floors and frequently place their hands or objects in their mouths, are particularly vulnerable in such environments^6^. As a result, the quality of housing and household infrastructure plays a critical role in determining children’s exposure to infectious diseases.

**Fig. 1:**
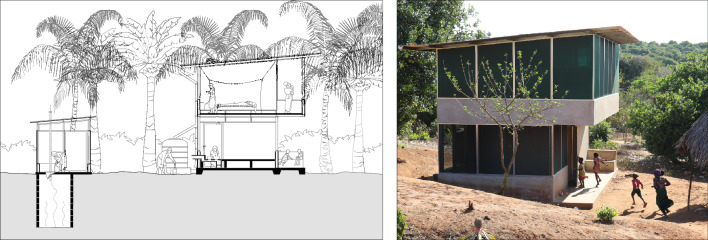
Star Home cross-section and frontal views (additional views of traditional homes and Star Homes can be found in extended data). The key features of a Star Home are as follows: (1) building orientated to provide optimal shading throughout the day to keep the house cooler at night (→ indoor climate); (2) lightweight and durable roof with partially closed eaves to reduce the entry of malaria vectors (→ malaria, indoor climate); (3) facade and openings screened to reduce insect entry while assuring airflow (→ malaria, indoor climate); (4) raised concrete ground floor that can be easily cleaned and increases hygiene and reduces the risk of enteric and soil-transmitted infections (→ diarrheal diseases); (5) a screened indoor cooking area with the means to remove smoke to reduce indoor pollution (→ respiratory infections); (6) a protected, lockable, ground floor storage area to reduce rodent infestations and provide a sense of security (→ safety); (7) sleeping areas with bednets raised to the first floor, improving airflow and comfort while reducing mosquito abundance (→ malaria, indoor climate); (8) a water harvesting system that allows the collection of rainwater from the roof, filtering and covered storage (→ diarrheal diseases); (9) an outdoor fly-proof latrine (→ diarrheal diseases); and (10) solar power providing electric light at night (→ safety).

In 2017, we conducted a pilot study in Magoda, Tanga region, Tanzania, to evaluate the benefits of innovative housing components, such as elevated bedrooms, enhanced ventilation, reduced smoke exposure, sufficient water supply and sanitation^14^. The study focused on indoor climate and mosquito abundance in elevated single-story or double-story homes constructed from bamboo, timber or shadenet materials and the community’s receptiveness to these designs. The double-story, shadenet housing received positive feedback from the community, showed evidence of a 96% reduction in indoor mosquito abundance compared to outdoors and was 2.3 °C cooler compared to unaltered reference houses. Empirical data from this study were used in computational fluid dynamics models to analyze indoor climate data, and to optimize passive cooling for comfort and increased bednet use at night. Further experimental studies in The Gambia showed that elevating a bedroom and having large, screened windows in the bedroom can reduce indoor mosquito entry^15,16^. The results from Tanzania and The Gambia helped to inform the design of the ‘Star Home’, which is the intervention in the trial described here (Fig. 1).

We hypothesized that a reduction of vectors, provision of safe water, improved sanitation and better air exchange with reduced smoke would result in a reduction of the three childhood diseases: malaria, ARIs and diarrheal diseases. To test this hypothesis, we carried out a 3-year cluster (household) randomized controlled trial of Star Homes in the Mtwara region in southern Tanzania, an area with a high malaria prevalence and high coverage of insecticide-treated nets. The primary outcome in this study was the relative risk of malaria among children under 13 years of age living in Star Homes compared to children living in traditional homes. Secondary endpoints included the incidence of diarrhea and ARIs and growth in children under 5 years of age.

## Results

### Assembly and disposition of study participants

**Fig. 2:**
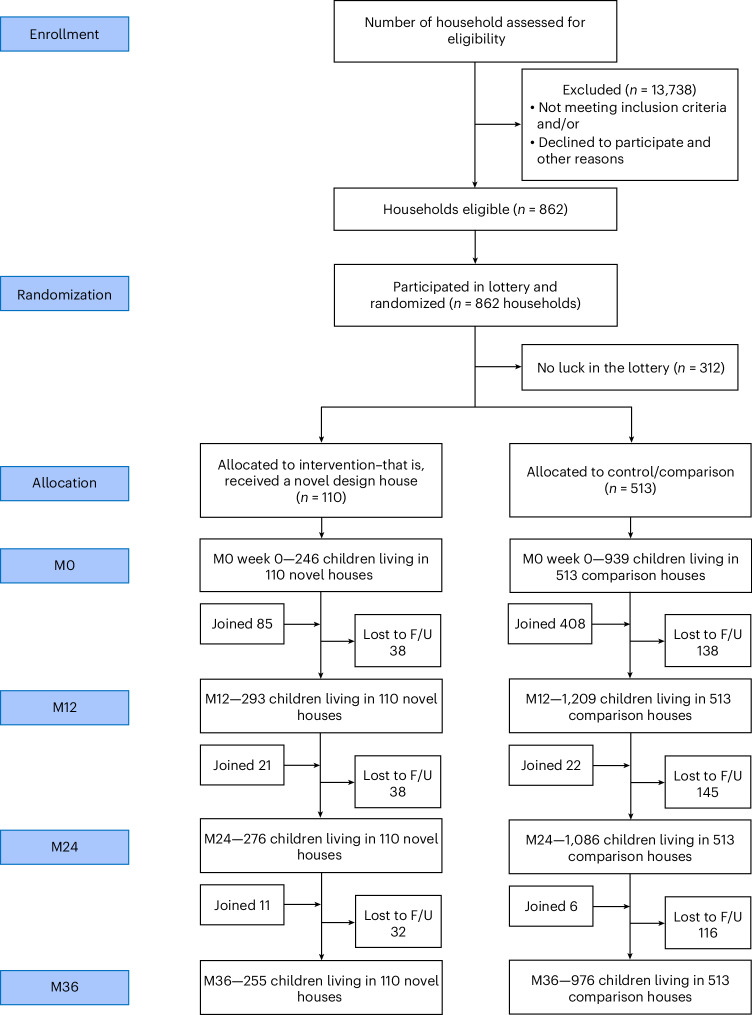
Trial profile. Profile of the dynamic study cohort indicates the recruitment of the study cohort. F/U, follow-up; M, month.

The construction of 110 Star Homes was completed in June 2021, and the participants moved into their new homes between June and December 2021. In January 2022, 247 children in 110 Star Homes and 936 children in 513 traditional homes were recruited into the trial cohort. The baseline characteristics of children in Star Homes and in traditional homes were similar (Table 1). Subsequently, participants who moved away and children who passed the age threshold left the cohort, and eligible newcomers and newborns entered the cohort, providing a constant replacement in this dynamic cohort (Fig. 2). Clinical surveillance was conducted over 36 months (4 January 2022 to 31 December 2024) through weekly home visits by trained field workers. By the end of the first year, the number of participants had increased to 293 in Star Homes and to 1,209 in traditional homes. The amount of time each child participated in the cohort was calculated in terms of person-years. During 2024, the last year of the study, weekly early morning visits found that 98% (6,356/6,518) of study children in Star Homes and 98% (21,712/22,197) of study children in traditional control homes were sleeping under bednets.

**Table 1:** Baseline characteristics of the participants by type of house design

	Star Homes (*n* = 247 participants)	Traditional houses (*n* = 936 participants)	Total (1,183 participants)
	*n* (%)	*n* (%)	*n* (%)
Sex			
Female	118 (48)	441 (47)	559 (47)
Age group (years)			
0−5	74 (30)	283 (30)	357 (30)
6−12	143 (58)	551 (59)	694 (59)
≥13	30 (12)	102 (11)	132 (11)
Informant			
Mother or primary caregiver	109 (44)	479 (51)	588 (50)
Father	84 (34)	308 (33)	392 (33)
Other family member	17 (7)	68 (7)	85 (7)
Other	37 (15)	81 (9)	118 (10)
Family living in the same house	240 (97)	862 (92)	1,102 (93)
The child missed school in the past 1 week	24 (10)	94 (10)	118 (10)
Child health on day 0			
Fever ≥37.5 °C	3 (1.2)	1 (0.1)	4 (0.3)
mRDT positive	101 (36.6)	423 (38.2)	524 (37.9)
Diarrhea, yes	5 (2.0)	10 (1.1)	15 (1.3)
Cough, yes	6 (2)	31 (3)	37 (3)

### Childhood malaria incidence

**Fig. 3:**
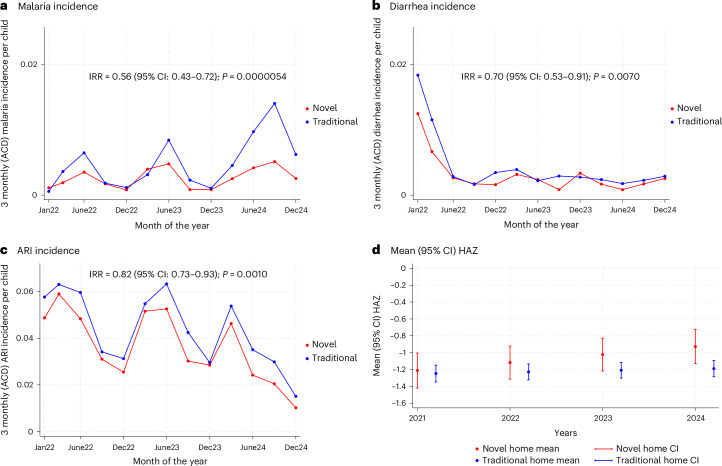
Impact of Star Homes on child health. **a**, Malaria episodes were 44% lower in children living in Star Homes than those living in traditional homes (IRR = 0.56 (95% CI: 0.43−0.72), *P* = 0.0000054). **b**, Diarrhea incidence was 30% lower in children in Star Homes than those living in traditional homes (IRR = 0.70 (95% CI: 0.53−0.91), *P* = 0.0070). **c**, Incidence of ARIs was 18% lower in children living in Star Homes than those living in traditional homes. Malaria, diarrhea and ARI incidence 2022–2024 by ACD, mean (95% CI) of HAZ (mixed-effect model for changes over time of the *z*-scores, accounting for clustering) (**d**). HAZ: In traditional homes, 221, 305, 338 and 346 participants, respectively, for years 2021−2024 were included in deriving HAZ statistics, whereas, in Star Homes, 52, 69, 76 and 74 participants, respectively, for years 2021−2024 were included in deriving HAZ statistics. Two-sided *z*-tests from the GEE Poisson models were performed, and there were no multiple comparisons, hence no adjustment for multiplicity. See also Extended Data Table 2.

**Extended Data Fig. 1:**
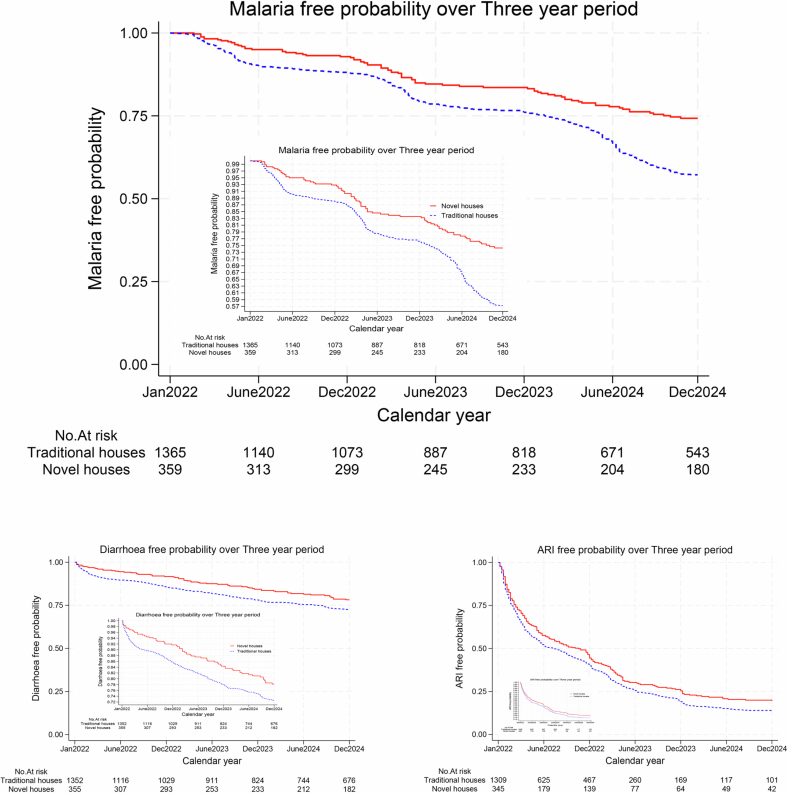
Malaria, diarrhoea, and ARI incidence. Malaria, diarrhoea, and ARI incidence 2022 – 24 by active case detection ACD, Kaplan Meyer survival analysis.

The primary outcome of the trial was malaria cases over 3 yearsʼ follow-up. The IRR of malaria cases detected by active case detection (ACD) was 0.56 (95% CI: 0.43−0.72, *P* < 0.0001), indicating a 44% reduction in the malaria incidence for children in Star Homes compared to those in traditional homes (Table 2 and Fig. 3). The malaria incidence peaked every year between May and June corresponding to the rainy season. The malaria season was highest and longest during the last year of surveillance, 2024 (Supplementary Fig. 1). The Kaplan−Meier survival analysis showed a higher malaria-free probability in children in Star Homes than in children in traditional homes, with the gap widening over time (Extended Data Fig. 1). In 2022, the malaria IRR by ACD was 0.64 (95% CI: 0.42−0.97, *P* = 0.034); in 2023, the IRR was 0.76 (95% CI: 0.51−1.12, *P* = 0.17); and in 2024, the IRR was 0.44 (95% CI: 0.31−0.63, *P* < 0.0001). The analysis of malaria cases detected by passive case detection (PCD) was a secondary outcome and showed a similar pattern with smaller effect sizes (Supplementary Figs. 2 and 3).

**Table 2:** Efficacy findings

Outcomes		Univariate model		Multivariable model	
		IRR (95% CI)	*P* value	IRR (95% CI)	*P* value
Malaria					
ACD:	Traditional home	REF	REF	REF	REF
	Star Home	0.56 (0.43−0.72)	<0.0001	0.56 (0.43−0.72)	<0.0001
PCD:	Traditional home	REF	REF	REF	REF
	Star Home	0.86 (0.75−0.98)	0.022	0.86 (0.75−0.98)	0.022
Diarrhea					
ACD:	Traditional home	REF	REF	REF	REF
	Star Home	0.73 (0.56−0.96)	0.024	0.70 (0.53−0.91)	0.0070
PCD:	Traditional home	REF	REF	REF	REF
	Star Home	0.87 (0.60−1.26)	0.45	0.84 (0.59−1.20)	0.34
ARIs					
ACD:	Traditional home	REF	REF	REF	REF
	Star Home	0.82 (0.72−0.93)	0.0030	0.82 (0.73−0.93)	0.0010
PCD:	Traditional home	REF	REF	REF	REF
	Star Home	0.92 (0.78−1.09)	0.36	0.91 (0.78−1.07)	0.24

IRRs comparing malaria, ARIs and diarrhea incidence between house designs over a 3-year period in children <13 years using a GEE Poisson model that accounts for clustering of individuals within households. Two-sided *z*-tests from the GEE Poisson model were performed. In the absence of multiple comparisons, no adjustment for multiplicity was performed. REF, reference group.

### *Plasmodium falciparum* parasitemia in children (secondary outcome)

**Extended Data Fig. 2:**
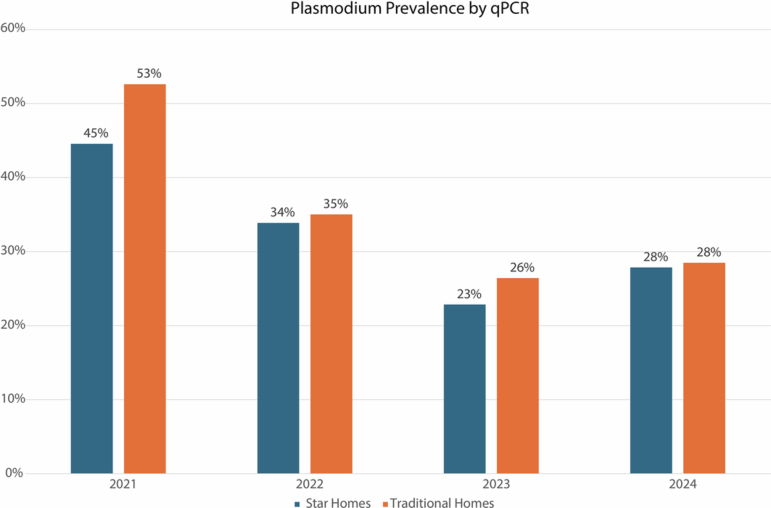
Plasmodium prevalence detected by qPCR. The cross-sectional malaria parasitaemia surveys analysed using qPCR showed a significant decrease in the prevalence of *Plasmodium* infections over the study period from 53% to 28% (p < 0.0001) among children residing in traditional homes, and from 45% to 28% (p = 0.008) among those in the Star Homes. There was no significant difference in *Plasmodium* prevalence between the study arms throughout the study years.

Cross-sectional *Plasmodium falciparum* infection surveys using quantitative polymerase chain reaction (PCR) showed a significant decrease in the prevalence of *Plasmodium* infections over the study period from 53% to 28% (*P* < 0.0001) among children residing in traditional homes and from 45% to 28% (*P* = 0.008) among those residing in Star Homes. No significant difference was observed in *Plasmodium* prevalence between the study arms throughout the study years (Extended Data Fig. 2).

### Diarrhea and ARIs (secondary outcomes)

Incidences of diarrheal diseases and ARIs were prespecified secondary endpoints. Children residing in Star Homes had a significantly lower diarrhea incidence than children residing in traditional homes (IRR by ACD = 0.70 (95% CI: 0.53−0.91), *P* = 0.0070). Using a Kaplan−Meier survival analysis, a higher diarrhea-free probability was also seen among children in Star Homes than in traditional homes (Extended Data Fig. 1). A disaggregated analysis by year showed a significantly lower diarrhea incidence for the first year, 2022, among the children in Star Homes compared to traditional homes (IRR = 0.65 (95% CI: 0.43−0.98), *P* = 0.041). This trend continued, but there was no significant difference in diarrhea incidence between the study arms during the following 2 years (Supplementary Fig. 4). Using data collected by PCD showed a similar diarrhea incidence pattern with smaller effect sizes (Supplementary Figs. 5 and 6).

The incidence rate of ARI was 18% lower among children in Star Homes compared to children in traditional homes using data collected by ACD (IRR = 0.82, (95% CI: 0.73−0.93), *P* = 0.0010). The Kaplan−Meier survival analysis illustrates the ARI-free probability over 3 years (Extended Data Fig. 1). No significant difference was observed in the incidence of ARI in the first 2 years; however, in 2024, the IRR was reduced by 25% (IRR = 0.75 (95% CI: 0.63−0.90), *P* = 0.0020; Supplementary Fig. 7). The analysis of the data collected by PCD showed a reduced effect size compared to ACD (Supplementary Figs. 8 and 9).

### The impact on growth (secondary outcome)

**Extended Data Fig. 3:**
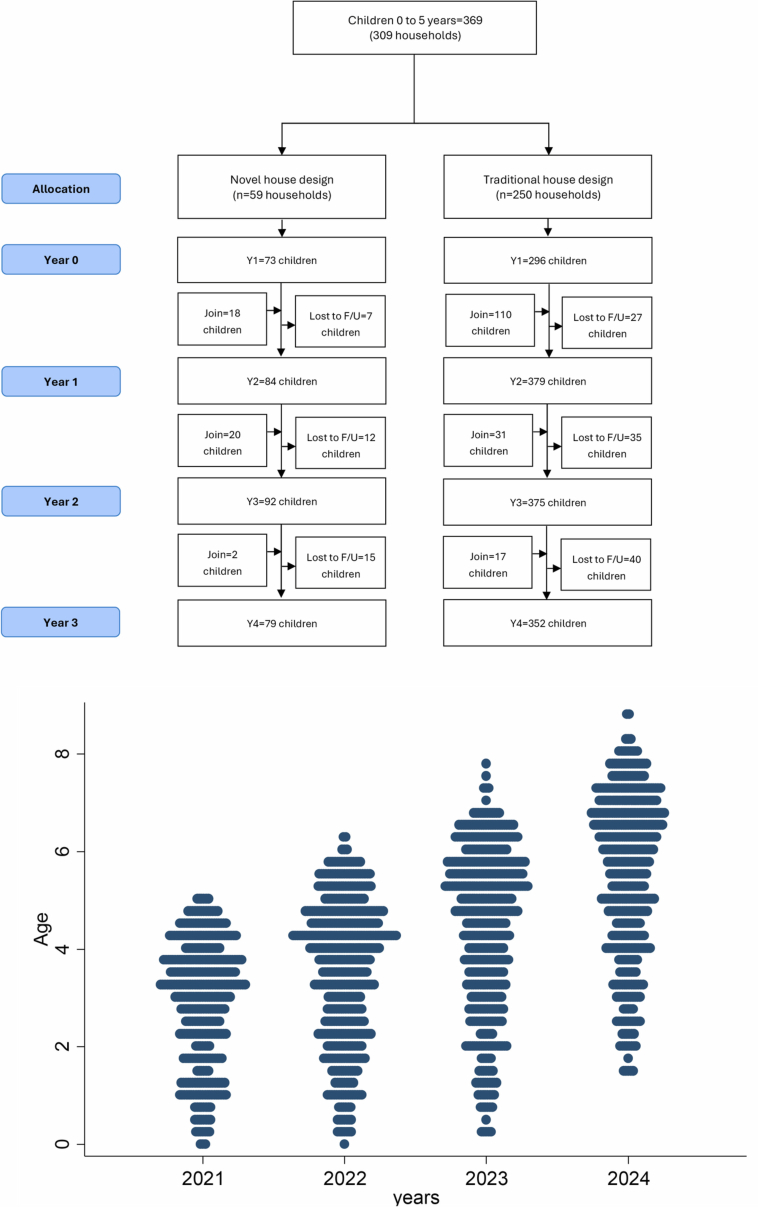
Anthropometry, assembly of participants. The assembly of a nested, dynamic cohort for anthropometric measurements is shown at top. *Age changes in the anthropometry cohort are shown underneath*.

Of the trial participants, 369 (31.2%) were under 5 years of age and participated in a nested, dynamic, anthropometric cohort, with 73 (19.8%) children in Star Homes and 296 (80.2%) children in traditional homes (Extended Data Fig. 3). At the end of the study period, 79 children in Star Homes and 352 children in traditional homes participated in the anthropometric component of the trial. The characteristics of the children participating in the baseline anthropometric survey were similar in both study arms (Extended Data Table 1). The median (range) age of children increased from 3.18 years (1.94−3.86) in 2021, to 3.82 years (2.35−4.68) in 2022, to 4.76 years (3.19−5.72) in 2023 and to 5.91 years (4.34−6.88) in 2024. The overall median (interquartile range (IQR)) height-for-age *z*-score (HAZ) at baseline was −1.23 (−1.93 to −0.60), with 56 (20.1%) children who were stunted and 15 (5.4%) who were severely stunted. The HAZ of children living in Star Homes had a significantly steeper slope (0.095 (95% CI: 0.052−0.137), *P* < 0.0001) compared to children living in traditional homes (0.019 (95% CI: −0.001 to 0.040), *P* = 0.062) (Fig. 3 and Extended Data Table 2). The overall median (IQR) weight-for-age *z*-score (WAZ) at baseline was −0.95 (−1.69 to −0.31), with 49 (13.3%) children who were undernourished and 15 (4.1%) who were severely undernourished. The median (IQR) weight-for-height *z*-score (WHZ) at baseline was −0.28 (−0.94 to 0.35), including 22 (7.4%) children who had wasting and three (1.0%) who had severe wasting. No significant difference was detected for WAZ and WHZ between the study arms (Extended Data Table 2).

### Accidents and injuries (secondary outcomes)

During the 3-year study period, two Star Homes were destroyed. An explosion occurred in one home after the house owner accidentally placed a jerry can filled with kerosene on a burning cooking stove. Another house was destroyed by a falling tree. Both accidents occurred in the morning when no residents were indoors. Both destroyed Star Homes were repaired and continued to be used. Five control houses were destroyed after accidental or intentional fires ignited thatched roofs and burned to the ground.

Injuries detected in study participants during the study period included animal bites, transport injuries, cuts and burns. Injuries occurred in both study arms at similar rates (Table 3). There were 87 disease episodes that required hospitalization and occurred in both study arms at similar rates. Seven study participants died, one residing in a Star Home, the other six in traditional homes. The deaths included two in hospital (one with severe malaria and one with severe anemia) and five at home (one drowned, one died after being hit by a motorbike, one died of sequelae from hydrocephalus and two died of unknown causes). None of the deaths was related to the intervention.

**Table 3:** Injuries, severe diseases and deaths

Variable	Star Homes *n* = 42,736 person-weeks		Traditional homes *n* = 171,663 person- weeks			
	Injuries	Rate^a^	Injuries	Rate^a^	IRR^b^ (95% CI)	*P* value
Injuries						
Animal bite	32/42,736	7	163/171,663	10	0.86 (0.60−1.22)	0.39
Transport injuries	18/42,736	4	82/171,663	5	0.95 (0.58−1.56)	0.85
Struck by an object	127/42,736	30	662/171,663	39	0.83 (0.68−1.02)	0.072
Cuts	50/42,736	12	275/171,663	16	0.80 (0.59−1.08)	0.15
Burns	32/42,736	7	155/171,663	9	0.91 (0.61−1.36)	0.65
Falls	89/42,736	21	391/171,663	23	0.98 (0.78−1.24)	0.86
Severe disease, deaths						
Severe disease resulting in hospitalization	23/43,113	5	64/173,582	4	1.46 (0.93−2.31)	0.10
Deaths (in hospitals)^c^	0/43,113	0	2/173,582	0	Not applicable	
Deaths (at home)^d^	1/42,736	0	4/171,663	0	Not applicable	

^a^ Episodes per 10,000 person-weeks

^b^ IRR adjusted for age, sex and seasonality. The full results that show the univariate and multivariable analysis models are provided in the supplementary materials.

^c^Causes of deaths: severe anemia and severe malaria

^d^ Causes of deaths: drowned, road traffic accident, congenital hydrocephalus and unknown (2)

A two-sided *z*-test from the GEE Poisson model was performed, and there were no multiple comparisons, hence no adjustment for multiplicity.

### Carbon footprint and economics of Star Homes (exploratory outcomes)

**Extended Data Fig. 4:**
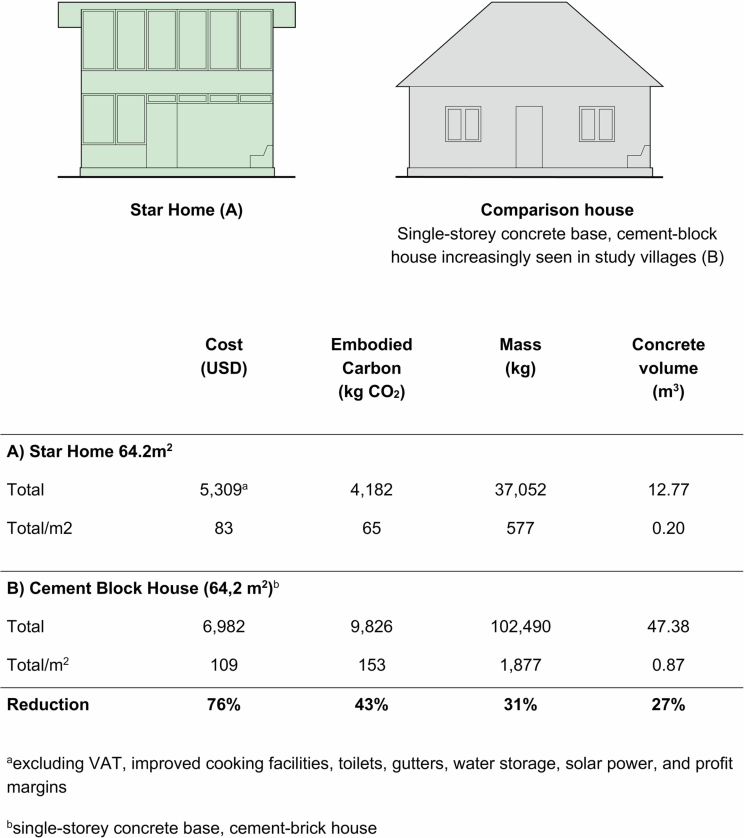
Comparison Star Home and cement-brick house. The Star Home costs 24% less than a cement-brick house, requires 57% less embodied carbon, weighs 69% less, and requires 73% less concrete. The houses are compared on the main construction alone; toilets, gutters, water storage and solar power installations are not included as they will be similar. The signed contract for 110 The signed contract for 110 star houses constructed by a professional contractor, Eco-homes, Dar es Salaam, was USD 969,980 for 110 houses or USD 8,818 per house including VAT, improved cooking facilities, toilets, gutters, water storage and solar power. The houses were constructed in 3 d BIM software, Archicad version 28.1.1 (Graphisoft, Budapest, Hungary). All quantities were extracted directly from Archicad. Embodied carbon was estimated on standards from the German standard ÖKOBAUDAT https://www.oekobaudat.de/en.html.

The carbon footprint of a Star Home is smaller than a single-story concrete base, cement block house—the preferred upgrade from a thatched wattle and daub construction in rural Tanzania (Extended Data Fig. 4). The Star Home weighs 69% less than such a cement block house, requires 73% less concrete and has 57% less embodied carbon, and the building materials cost 24% less (Supplementary Tables 1 and 2). A single-story concrete base, cement block house costs $109 per m^2^ compared to $118 per m^2^ for a Star Home. When the steel construction is replaced with timber and bamboo, the construction costs for a concrete base, double-story, timber, bamboo, shadenet house could decrease to less than $90 per m^2^, which is at least 20% cheaper than the average modern house being constructed in sub-Saharan Africa today. A preliminary order-of-magnitude assessment suggests an estimated four-fold return on investment over a 50-year timeframe, as detailed in Extended Data Table 3 and economic assessment in the supplementary materials.

## Discussion

We provide support for the hypothesis that improved housing can reduce the burden of three lethal diseases of childhood and improve a child’s growth, leading to a reduction in stunting. After 36 months, children living in Star Homes had a significantly reduced risk of malaria (44% reduction), diarrhea (27% reduction) and ARIs (18% reduction) compared to children living in traditional mud and thatched-roof homes. Children living in Star Homes had a greater increase in HAZ compared to children living in traditional homes. Injuries, hospitalizations and deaths were similarly distributed between the two study arms. Our finding of a reduction in malaria incidence was supported by our entomological surveillance, which found 51% fewer house-entering *Anopheles gambiae*, the principal African malaria vector, in Star Homes compared to traditional homes^17^. Our results are consistent with earlier observational studies in sub-Saharan Africa that found a 47% reduced likelihood of malaria associated with improved housing^18^.

Randomized controlled trials to evaluate specific house modifications reported a similar trend with smaller effect sizes in malaria reduction^12^. The reduction in malaria incidence observed in our study changed over time. The effect size during the first year was smaller than in the final, third year. The malaria epidemiology also changed from year to year, with a larger and longer malaria season during the final, third year. It is reassuring that the benefits of improved housing were not reduced by the higher malaria endemicity and did not wane with time, as is the case with other interventions, such as vaccines. This observation may be explained by the period required by the study participants to make consistent, best use of their new home, such as sleeping in the upstairs bedroom under a bednet rather than in the lower indoor level or outdoors. The uptake of the new homes was initially unsatisfactory because of anxiety fueled by envy and rumors^19^. By the end of the trial period, apprehensions and rumors regarding Star Homes and the people living in them had subsided.

Data collected in healthcare centers through PCD showed similar trends with a reduced effect size. The most likely explanation for this observation is the incomplete and inconsistent use of reliable diagnostic tests by the healthcare providers in health centers. Fevers of various etiologies may have been misdiagnosed, resulting in random misclassification errors sufficient to dilute the impact estimates with a bias toward null^20^. The prevalence of *P. falciparum* infections was approximately halved over the study period, probably related to improved diagnosis and treatment of clinical malaria cases. We did not see any differential effect in the prevalence or density of *P. falciparum* infections detected by PCR from dried blood spots (DBSs). The absence of differences between study arms may be best explained by the persistence of subclinical infections that remained untreated during the study^21^.

In general, sanitation interventions tend to have a limited impact on diarrhea prevalence—15% in all ages in a large systematic review^22^. By contrast, our trial found a 30% reduction in diarrheal diseases in residents of Star Homes compared to residents of traditional homes. A fly-proof, easy-to-clean improved ventilated pit latrine situated away from the main house, the availability of safe water through rainwater harvesting and an elevated, easy-to-clean ground floor and cooking area may have all contributed to a reduced risk of diarrheal disease transmission, suggesting at least an additive effect. Entomological surveillance during the first 2 years of the study showed that there were 49% fewer *Chrysomya putoria* (the African latrine fly), a putative vector of numerous diarrheal pathogens, in Star Home kitchens than in traditional kitchens, with no flies emerging from the Star Home latrine compared to a mean of 4.2 flies per trap per day in traditional toilets^23^.

In a large meta-analysis, several improvements in housing conditions reduced the risk of upper respiratory tract infections by 18%^24^. This result is similar to the 18% reduction in ARI incidence found in our trial. Star Homes are designed to optimize cross-ventilation by cladding the building in an air-permeable shadenet. Second, Star Homes provide improved cooking stoves that divert smoke from indoor cooking fires outdoors in contrast to traditional homes where smoke from open fires is inhaled by household members. The corrugated iron roofs and concrete floors in Star Homes reduce the dust and allergen accumulation from thatched roofs and earth floors in traditional homes. The combination of decreased household air pollution with increased air exchange should reduce the risk of chronic airway irritation and the transmission of airborne pathogens.

An important positive effect of Star Homes was on the growth of young children. The impact on growth can be seen as an outcome on a final, multifactorial pathway to better health. Deficiencies in health and/or nutrition, irrespective of etiology, almost always have a negative impact on growth^25^. Other benefits from living in a Star Home included removing chores from household members, such as the constant rehabilitation and repair of the mud house. Rainwater harvesting removes the need to collect water from a remote source several times a day. Solar power allows indoor illumination that makes it more rewarding to stay indoors after sunset, perhaps providing a more nurturing environment for children and their parents. The absence of impact on weight is best explained by children in Star Homes catching up in linear growth while weight gain lags behind. In what is called ‘lean catch-up growth’, the child’s metabolism prioritizes skeletal and lean tissue growth once infection or inflammation is reduced^26^. Lean catch-up growth is more likely to occur during the early years of life, with adequate but not excessive nutrition and in physically active children—conditions present in the study.

The trial demonstrates how improved housing can protect children from disease. First, it is important to improve the climate comfort in African houses, relying on passive cooling rather than on air conditioning, which requires energy that is neither available nor affordable^27^. After sunset, the upstairs space in the Star Home cools down rapidly, with optimized cross-ventilation ensuring a more comfortable climate during the night than in a traditional home and more comfortably allowing the use of bednets. During the day, the downstairs space remains cooler compared to more sun-exposed spaces. With rising temperatures, such passive cooling is becoming increasingly essential. Not adapting to increasing temperatures is not an option.

Our study had limitations. First, it was not possible to blind the study participants or research assistants to the study group. Because the research assistants in charge of data collection were aware of the study hypothesis, it is possible that research assistants preferentially reported negative outcomes from control homes and better outcomes from Star Homes. Such observer bias could have inflated the ACD results, which would help to explain the difference between the blinded PCD and unblinded ACD results. We do not think this was the case because our concomitantly conducted ‘bias-indicator study’ of the reported incidence of injuries showed similar results in both study groups, suggesting the absence of bias. Furthermore, the largest effect size was for malaria incidence, supported by the reduction in malaria vector abundance reported by an independent entomology team^17^. A second limitation was the limited diagnostic accuracy of the infections. *P. falciparum* infections were detected using the rapid diagnostic test (RDTs) available at the time in Tanzania. The resources required to set up quality-assured microscopy were not available. Diarrheal diseases and ARIs were diagnosed clinically without identification of the etiologic pathogen. This may have resulted in misclassification errors^20^, but because the field workers were trained to apply standardized methods, misclassifications were likely to occur at similar rates across both study arms. Third, the success of the intervention depends on its uptake. Children sleeping outside the Star Home cannot benefit from it. The study staff encouraged the use of the Star Home but could not prevent children from traveling or sleeping in the homes of friends or family. We mitigated this potential problem by having the field workers visit the homes early in the morning (around 5:00) to verify where the children were sleeping. Fourth, the study evaluated benefits exclusively for children under 13 years of age. Although it is reasonable to expect that older children and adult household members may also experience advantages from a more comfortable, cooler home environment and decreased disease transmission, these potential benefits were not captured by the trial. Finally, we can only demonstrate the benefits of one Star home within a setting that mainly consists of traditional homes. Children who live in Star Homes interact and play with those from traditional homes, which exposes them to potential infections and reduces some of the protection that Star Homes offer. If all children were to reside in better housing, the positive impact would likely be even greater.

The United Nations medium scenario suggests that the current population of Africa will expand from the current 1.5 billion to between 2.7 billion and 3.7 billion by 2070 (refs. ^28,29^). Considering the need to replace much of the current housing stock, hundreds of millions of housing units will be constructed in Africa in the coming decades. We show that small improvements in design, such as double-story buildings, cross-ventilation, mosquito screening, self-closing doors, clean water harvesting, improved pit latrines and improved cooking stoves, are likely to make a major health impact. Although the Star Home may not be the final product, it provides innovative ideas for how to build better, healthier homes for the control of multiple diseases. We hope that our present study will stimulate further innovation in this field with those working in the built environment with local communities to construct healthier homes for rural people in low- and middle-income countries—an approach supported by Sustainable Development Goals (Supplementary Table 3). Simple improvements in housing can have profound impacts on improving public health.

## Methods

### Study design and participants

**Extended Data Fig. 5:**
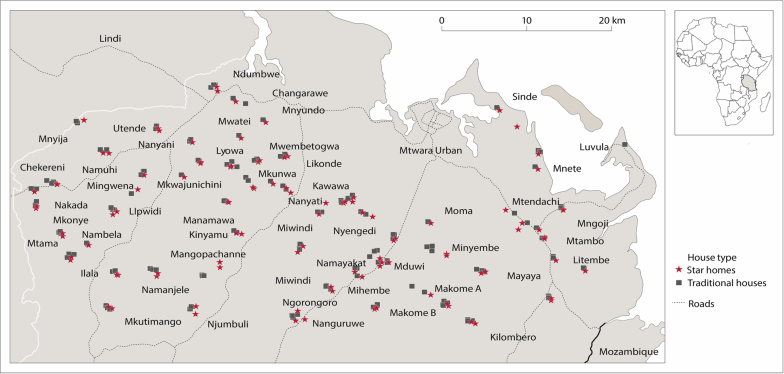
Map of the study area. Mtwara district was selected as study site based on a) high malaria prevalence b) hot, humid climate, and c) relatively limited vector-control interventions and medical research activities compared with northern Tanzania^27^. Villages were eligible for inclusion if they had: 1) a population exceeding 100 households, 2) road accessibility throughout the year, 3) no connection to the national electricity grid, 4) no municipal water supply, and 5) high malaria prevalence.

This is an open-label, household-cluster, randomized controlled trial using a generalized, randomized, complete block design, with the village serving as a block, conducted from January 2022 to December 2024. The study protocol was published in 2022 (ref. ^26^). The trial was conducted in rural Mtwara, Tanzania, bordering with Mozambique (Extended Data Fig. 5). Mtwara is the region with the second highest malaria prevalence in Tanzania in 2023 (ref. ^27^). In total, 67.3% of the population of Mtwara has access to insecticide-treated nets (ITNs)^27^. Prior to randomization, we surveyed 14,600 households in 70 villages to assess eligibility to participate in a house lottery. The inclusion criteria were as follows: (1) informed consent to participate in the study, including active surveillance for 3 years; (2) owning sufficient land for the new house; (3) having three children under 13 years of age in the household; and (4) residing in a traditional home characterized by mud walls, thatched roof and an earthen floor, with a pit latrine, no electricity supply and no piped water from the municipality (Supplementary Figs. 1–3). Of the 14,600 households surveyed, 862 were eligible to participate in the lottery.

### Randomization

Participants were randomly assigned to study groups using a lottery-based allocation procedure over two rounds. In each village, the names of eligible heads of households were written on slips of paper, each placed in an individual envelope. All envelopes were put in a transparent bucket and mixed by a village volunteer schoolchild, who, in public, drew between one and three envelopes depending on the village’s size. The winners became owners of Star Homes. The remaining envelopes were included in the next round to select 4−12 additional envelopes. The winners of the second round were invited to serve as comparison households. A dynamic cohort of children residing in 110 Star Homes (intervention) and in 513 traditional African homes (control) was recruited.

### Ethics approval

This report follows the CONSORT 2025 extended guidelines^28^. The trial protocol was approved by the Tanzanian National Institute for Medical Research (NIMR/HQ/R.8a/Vol.IX/3695) and the Oxford Tropical Research Ethics Committee (OxTREC 533-20). Written informed consent was obtained from the parents or legal guardians of all participating children.

### Interventions

**Extended Data Fig. 6:**
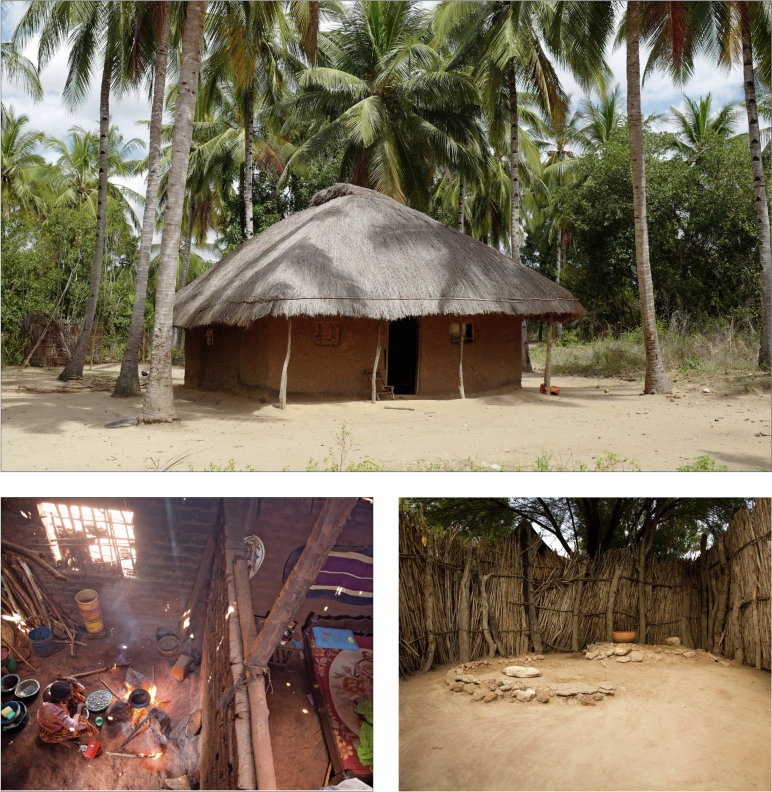
Traditional Home. Exterior of a newly built, traditional home, a wattle and daub construction in Mtwara, Tanzania (top), Interior of a traditional home with indoor cooking (bottom left), Traditional outdoor latrine and space for washing/showers (bottom right).

**Extended Data Fig. 7:**
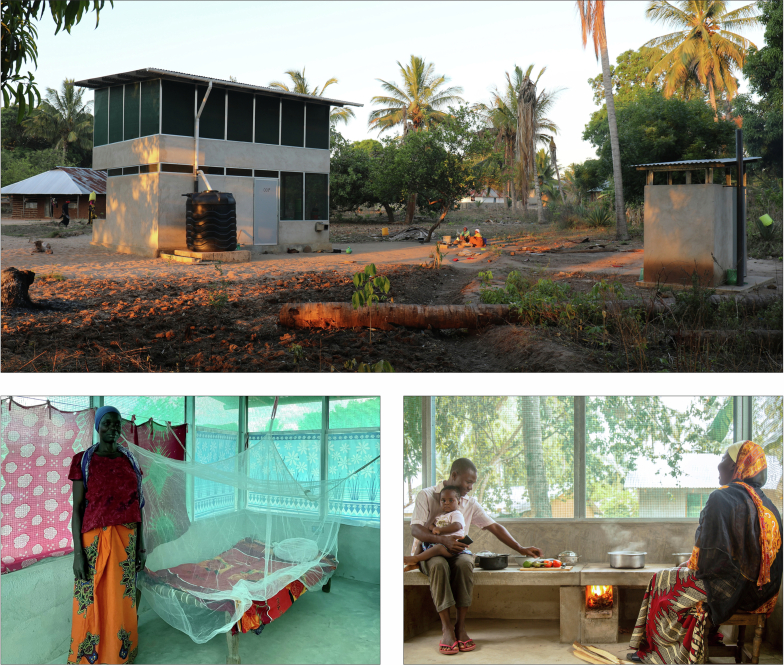
Star Home. Star Home, exterior with rain water harvesting system gutters and water tank. The ventilated pit latrine is on the right (top), interior upstairs, furnished (bottom left), downstairs, kitchen (bottom right).

The Star Home is a double-story, light gauge steel construction (Fig. 1 and Extended Data Figs. 6 and 7). To reduce mosquito ingress, the entire building, including the upstairs sleeping room, is surrounded by an air-permeable, polyethylene shadenet facade that optimizes cross-ventilation to increase climate comfort and use of ITNs and minimizes indoor carbon dioxide concentration, used by mosquitoes for host location. The addition of self-closing doors reduces mosquito house entry. Components targeted at reducing diarrheal disease include: (1) the ground floor and work surfaces in the kitchen are concrete and are slightly elevated to facilitate cleaning; (2) a secure, rodent-proof storage room on the ground story prevents rodent entry; (3) a shadenet facade and self-closing doors reduce indoor fly entry; (4) rainwater collected from the corrugated iron roof is piped into a 2,000-liter polyethylene storage tank where it is filtered and collected, providing copious amounts of clean water for drinking and washing; and (5) a ventilated, fly-proof pit latrine stands separate from the house to reduce the production of flies and mosquitoes. To prevent respiratory diseases, the shadenet walls maximize ventilation in the entire house. An improved cooking stove minimizes household air pollution^30^. Other features to improve the home include solar panels on the roof, which provide electricity for phone charging and light at night.

Star Homes were constructed over 25 months from 1 June 2019 to 30 June 2021. All homeowners had relocated to their new homes over 6 months by 31 December 2021. A maximum of three Star Homes were constructed in each study village to mitigate the risk of mosquito diversion and significant alterations to the village appearance and ambiance. In 12 villages, one Star Home was built; in 46 villages, two Star Homes were built; and in two villages, three Star Homes were built (Supplementary Fig. 2). The prefabricated components of the Star Homes were manufactured in Dar es Salaam, Tanzania (Ecohomes Company Limited), and were shipped to Mtwara by road. Once the concrete foundation was in place, the assembly of a Star Home took 3−4 days. The price the study paid the construction company for a Star Home was $8,818, or $118 per m^2^. A comparison of material costs for house types was based on houses constructed in 3D BIM software, Archicad version 28.1.1 (Graphisoft). All quantities were extracted directly from Archicad. Embodied carbon was estimated on standards from the German standard ÖKOBAUDAT (https://www.oekobaudat.de/en.html). A maintenance team was available during the study period to repair Star Homes as needed.

After the handover to the new owners, 11 homes remained unoccupied, and other study participants slept inconsistently in their new homes^31^. A multipronged approach was undertaken to understand and overcome the hesitancy of homeowners to make full use of the new homes, which included qualitative research, embedding of research assistants with study households, sport ‘bonanzas’ including football games and a radio drama^19^. The new homeowners who were formerly some of the economically most disadvantaged community members found themselves in a difficult situation when their neighbors, who were envious after losing the lottery, fueled distrust toward the research team^19^. By the end of December 2021, most barriers had been overcome, and all study participants were consistently sleeping in their new homes. The extensive qualitative findings collected during the trial were previously published^19,31,32^.

Every study household (intervention and control) was provided with national health insurance, two mattresses and blankets. ITNs were distributed free of cost at the beginning of the study, as this is current best practice. To improve ITN use, children were told about the benefits of the nets and the importance of sleeping in the upstairs rooms. Incentives such as toys, books, shoes and stationery were awarded to children who adhered to the guidelines and slept under ITNs, as verified by the research assistants (see also below). The distribution of initiatives and incentives was identical in Star Homes and traditional homes. At the end of the trial, in February 2025, every control household received building materials, consisting of a 2,000-liter water tank and 20 corrugated iron sheets.

### Procedures

Extensive community engagement activities were conducted prior to and throughout the study to explain the study procedures and encourage full participation^19^. The first census was conducted in 2018 and was updated in 2021. Baseline demographic and clinical data were collected from all children under 13 years of age by January 2022 (no lower age limit) or upon their enrollment in the study. Each study child was issued a photographic identification card to facilitate identification during follow-up.

Episodes of malaria, diarrheal diseases and respiratory tract infections were detected by ACD and PCD, starting in January 2022. ACD was conducted by 22 trained research assistants who visited the homes every week in pairs, one of whom was medically trained. During the weekly visits, the research assistants made an observational assessment of bednet usage early in the morning while the children were still asleep and inquired about illness or injury during the preceding week. A malaria episode was defined as presence or history of fever with an axillary temperature of 37.5 °C or higher and a positive malaria rapid diagnostic test (mRDT) using Paracheck (Orchid Biomedical Systems), ParaHIT (ARKRAY Healthcare Pvt. Ltd.) or Bioline Malaria Ag P.f/Pan (depending on in-country availability). Diarrhea was defined by three or more loose stools within 24 hours or one or more bloody loose bowel movements. Participants presenting with fever or a history of fever in the previous 48 hours, combined with fast breathing (elevated respiratory rate for age and/or difficulty in breathing, or runny nose, or coughing), were documented as ARI cases^33^. Ill children were referred to the nearest health facility for management, with research assistants assuring access, typically dispensaries or health centers. A ‘bias-indicator study’ was conducted in the form of surveillance of injuries during the weekly visits by the research assistants, assuming that injuries occur at similar rates in both study arms. Data collected during ACD were entered directly into an electronic ACD study form in tablets and transmitted to a central server using custom-built software.

Treatment-seeking behavior of the study population was recorded in a study completed before disease surveillance started^34^. To capture participants visiting healthcare facilities, all relevant local healthcare providers were briefed about the project and were trained in completing a structured PCD study form on paper. Participants were encouraged to present their photographic identification cards during visits to healthcare facilities. The PCD forms were collected weekly from healthcare facilities by the research assistants and entered into a custom-built database. To ensure the accuracy of the data collected, routine checks were implemented, whereby a subset of entries was cross-verified with paper records at each healthcare center. Additionally, feedback loops were established with local healthcare providers to discuss data discrepancies and improve reporting processes. Training sessions were held bimonthly to refresh the knowledge of healthcare staff on data entry protocols and the importance of accurate information capture.

### Safety monitoring

The intervention consisted of structural improvements to homes and was considered minimal risk; therefore, no formal data and safety monitoring board was established. Safety monitoring was conducted through routine weekly household visits by trained research assistants throughout the study period. During these visits, field staff assessed household conditions and the well-being of participating children and caregivers and recorded any adverse events or safety concerns reported by participants or observed during the visit. Any adverse events potentially related to the intervention would have been documented and reported to the study investigators and the relevant ethics committees in accordance with the study protocol. No adverse events or safety concerns related to the housing intervention were identified during the trial.

### Protocol deviations

No deviations from the prespecified study protocol were identified during the conduct of the trial.

### PCR testing

Cross-sectional parasitemia surveys were conducted at baseline and annually after the rainy season (late May to early June) when *P. falciparum* prevalence peaks. All study children were invited for malaria testing consisting of an mRDT and a DBS. DBSs were packed in individual plastic bags with dehumidifiers, stored at −20 °C and transported to the Mahidol−Oxford Tropical Medicine Research Unit (MORU) in Bangkok, Thailand. DNA was extracted from DBSs using the QIAamp DNA Mini Kit (Qiagen) and quantified by absolute quantitative PCR targeting *Plasmodium* 18S rRNA, using a standard curve (20 to 20 million parasites per milliliter)^35^. Reactions were performed on a Rotor-Gene Q using the Quanti-Tect Multiplex PCR Kit with a cycle threshold cutoff of 40. Specificity was assessed with 100 negative control samples.

### Anthropometry

In October 2021, we obtained anthropometric measurements of study participants under 6 years of age. During the following annual visits, participants under 6 years of age were enrolled into the nested, dynamic cohort. Two trained study staff measured the weight and height of the children during each year of the study according to detailed standard operating procedures.

During each round of anthropometric measurements, the team comprising five study staff members (two who measured the children, two data recorders and a driver) visited the villages over a 2-week period according to a prepared schedule (4−7 villages per day, depending on the number of children and village location). The parents or guardians were requested to bring the children to the village office on their assigned day. The children were identified using their study identification cards, with the assistance of the village head and the study research assistant assigned to their village. If a parent or guardian was unable to bring their child, we visited their house or school. Measurements were recorded using Excel (Microsoft) on a laptop and separately on a tablet or phone using a custom-made data capture application. The two sets of data were compared in real time, and any discrepancies were resolved. The final dataset was verified using range and consistency checks.

The weight and height/length (for those 6 months of age and older) were measured as follows. Children over 2 years of age and able to stand were weighed in light clothes on a flat digital scale (seca, model 876). Each child was requested to remove his or her footwear and to stand in the middle of the scale, feet slightly apart, and to remain still until the weight appeared on the display. For younger children or those unable to stand, the mother’s weight was measured. Next, the scale was tared, and the mother was asked to carry the child while remaining on the scale to obtain the child’s weight. We recorded the weights to the nearest 0.1 kg. The heights of children 2 years and older and able to stand were measured using a stadiometer (seca, model 217). Recumbent lengths of children under 2 years of age were measured using a board (seca, model 417). We recorded the heights and lengths to the nearest 0.1 cm. During each measurement round, we gave each participating child a small treat and their parents a bar of soap. We aimed to include all the eligible study children in the four measurement rounds.

### Outcomes

The primary outcome was the incidence of *P. falciparum* malaria detected through ACD during weekly household visits. Secondary clinical outcomes included the incidence of *P. falciparum* malaria detected through PCD as well as the incidence of childhood diarrheal disease and ARIs, measured through both ACD and PCD. Incidence rates were expressed as the number of episodes per 1,000 person-years of observation, and differences between study groups were estimated using IRRs adjusted for intervention status, age, sex and season. Parasitological outcomes included *P. falciparum* prevalence measured during cross-sectional malaria surveys using molecular diagnostics. Child growth and nutritional status were assessed in a nested anthropometric cohort of children under 5 years of age. Anthropometric outcomes included HAZ, WAZ and WHZ. Economic outcomes included an assessment of the cost and carbon footprint of Star Homes compared to conventional rural housing as well as an exploratory cost–benefit analysis estimating the long-term return on investment associated with the intervention.

### Building parameters

Trial homes were built using a standardized prefabricated system to ensure identical structures for the purposes of the randomized controlled trial. From the development and testing of the prototype, we learned that the core features responsible for the reduction in malaria, particularly the raised sleeping areas and large screened openings, are design elements rather than material-dependent innovations. The concept can be adapted to locally available materials and construction traditions, reducing reliance on specialized contractors. The trial homes were assembled by a company based in Dar es Salaam, Tanzania. With some training, the construction methodology can be implemented by local builders using familiar tools and techniques. The most frequently encountered maintenance issue was torn nets, which can be repaired or replaced with supplies available in local hardware shops. This supports the feasibility of long-term upkeep in a rural African setting. Although the exact version tested in the trial may not represent the final form or forms, the underlying design principles are robust, flexible and suitable for adaptation. We, therefore, recommend that future scale-up efforts focus on translating these design parameters into locally appropriate, cost-sensitive construction solutions that align with community preferences and available skill sets.

Key building parameters, mass, building costs, concrete volume and embodied carbon were compared between a Star Home (A) and a single-story concrete base, cement block house (B) of the same size—the preferred upgrade from a wattle and daub construction (mud hut) in rural Tanzania.

### Economic assessment

We conducted a preliminary order-of-magnitude economic assessment to estimate the internal rate of return of capital invested in a Star Home over a 50-year time horizon, reflecting the expected structural lifespan of permanent residential buildings constructed with reinforced foundations and durable superstructures. The analysis adopted a societal perspective and included construction and maintenance costs as well as economic benefits arising from improvements in health outcomes, energy efficiency and household infrastructure. Construction costs were based on the signed contract for 110 Star Homes built by a professional contractor in Dar es Salaam, corresponding to a total cost of $969,980 ($8,818 per house, including VAT). The construction package included improved cooking facilities, sanitation (ventilated pit latrine), rainwater harvesting with gutters and storage tanks and solar power for lighting. Annual maintenance costs were estimated using the conventional 1% rule, assuming that 1% of the initial construction cost is invested annually in maintenance. Health-related economic benefits were estimated from the observed protective efficacy of Star Homes against common childhood diseases. Reductions in malaria (44%), diarrheal disease (30%) and ARIs (18%) were translated into economic benefits using published estimates of treatment costs, household expenditures, productivity losses and the value of statistical life^36,37^. Health system savings included the cost of treatment per case; household savings included out-of-pocket expenditures and transport costs; and productivity gains reflected lost workdays due to illness among adults or caregivers. Additional benefits included improved child growth and its associated lifetime productivity gains, reductions in indoor heat exposure, improved access to water and sanitation, reduced household energy expenditures through solar lighting, improved cooking efficiency and health benefits associated with reductions in indoor air pollution^38–40^. Cost savings were also estimated relative to the typical lifecycle costs of traditional rural housing structures that require frequent repair or replacement. Population-level disease incidence estimates were derived from national and international surveillance datasets, including the World Health Organization Global Health Observatory^41^. Wage estimates were obtained from publicly available labor market statistics from the Tanzania National Bureau of Statistics^42,43^. Household size was assumed to be five people. All costs and benefits were expressed in US dollars and projected over a 50-year period to estimate the overall return on investment. Detailed parameter values and intermediate calculations are provided in the supplementary materials.

### Ethics and inclusion statement

The project was developed through a long-established partnership among CSK Research Solutions (a Tanzanian research organization), the Royal Danish Academy of Architecture and the MORU in Bangkok, Thailand. This team previously conducted a pilot study of novel design houses in Magoda, Tanzania. For the multidisciplinary project in Mtwara, additional research groups joined. Entomologists from Tanzania’s Ifakara Health Institute and the University of Durham, UK, provided support for the project’s entomological research. Qualitative research played an essential role and was carried out by social scientists from Tanzania, The Netherlands and Nepal. The social science team designed the qualitative studies with contributions from the wider research group, adapting the approach as the study progressed (S.M., manuscript in preparation).

Residents were engaged at several stages of the design process. Responses to prototypes built in Magoda were studied within the first year of occupation^14^. Through qualitative interviews, residents and neighbors gave feedback on six house types, varying in story and cladding, to identify their preferred features. Investigators from CSK Research Solutions, the Royal Danish Academy and MORU continue to visit the households annually, asking about durability and performance during inspections and walk-throughs, which has generated suggestions for improving building performance.

Feedback from residents led to changes in the house design. In Mtwara, the Star Homes feature a covered porch with a ‘Swahili bench’, providing a semiprivate area for sitting, socializing and daily tasks such as food preparation and handicrafts. The layout follows local Swahili traditions, placing cooking areas in more private sections and arranging sleeping spaces deeper within the home or upstairs in the Star Home—to keep them away from the entrance and reduce exposure to mosquitoes, which are more common at ground level. To improve security, lockable storage spaces were added. Before building 110 Star Homes, three additional prototypes were constructed and evaluated in Mtwara. Residents gave feedback on house design, latrines, stoves and kitchen layout, leading to changes such as adjusting the facade for increased privacy.

The study data were entered using a locally developed system. Over several months in 2025, two investigators from Tanzania traveled to Bangkok to collaborate with MORU statisticians on analyzing the data. Two Tanzanian PhD/DPhil candidates, along with one Danish candidate, either have completed or are currently working toward advanced research degrees analyzing findings from the project.

For the purpose of estimating sample size, we made the assumption of 10 malaria episodes per 100 children per year in the control households, with a projected 30% decrease to seven malaria episodes per 100 children per year in the Star Homes^44^. To detect this difference with 80% statistical power and a 5% significance level, we determined that 105 households with three child-years of observation each would be required per group. Assuming that malaria was likely to have a lower incidence than diarrheal diseases and ARIs, the sample size calculated for malaria should be adequate for detecting significant differences in the incidence of all three diseases. To account for potential loss to follow-up, we enrolled 110 households into the intervention group. Given the necessity to adjust for clustering by both village and household during analysis, power simulations were conducted using a Poisson random effects model to determine the optimal ratio of traditional to Star Home households that would ensure adequate statistical power, assuming a coefficient of variation of 0.25 (ref. ^45^). For every intervention household, a minimum of four control households was deemed necessary. Due to the limited information available in the existing literature regarding intracluster correlations (ICCs) at the village and household levels, we opted for a conservative estimate of a baseline ICC of 0.6 (ref. ^46^).

### Statistical analysis

The data analysis adhered to a predetermined analytical plan established prior to the completion of the study. Malaria incidence data, serving as the primary parasitological endpoint, were analyzed using generalized estimating equation (GEE) Poisson models to detect differences in incidence rates between the two arms, accounting for the repeated measurements within a house and the effect of year and seasonality (rainy or dry). The IRRs were estimated from the GEE Poisson models to compare malaria/diarrhea/ARI outcomes between the two study groups, accompanied by the corresponding 95% CIs. Initially, univariate GEE Poisson models were fitted, followed by the implementation of multivariable GEE Poisson models.

A quarterly incidence line plot was generated to compare the occurrence of malaria, diarrheal diseases and ARIs over a 3-year period across two study arms. Participants who moved away or completed the full 3 years without experiencing any of the specified diseases were censored at their last known time or at the end of the observation period, respectively. New participants were considered disease free at the study’s onset until the detection of any infection. Those presenting with malaria, diarrheal diseases or ARIs at baseline were excluded from the analysis, having already exhibited the outcomes of interest prior to the intervention. This study cohort is dynamic, with later joiners assumed uninfected at the surveillance’s commencement until an event occurs. Baseline variables, categorized as such, were summarized using counts and percentages.

To give a visual impression, Kaplan−Meier plot was used to summarize the probability of remaining disease free over the entire period as a complement to the detailed Poisson regression models.

We calculated WAZ, WHZ and HAZ. Undernutrition is defined as WAZ below –2 s.d.; wasting is defined as WHZ below –2 s.d.; and stunting is defined as HAZ below –2 s.d. Mixed-effects linear regression models were used to compare the continuous *z*-scores (anthropometric measurement summaries) obtained at the final round of each participant adjusted for participants’ baseline *z*-score as a covariate.

Significance tests were conducted at a 5% level of significance. Analyses were performed in Stata software (version 18). The statistical analysis plan is available upon reasonable request. The trial was registered with ClinicalTrials.gov (NCT04529434) on 27 August 2020.

### Reporting summary

Further information on research design is available in the Nature Portfolio Reporting Summary linked to this article.

## Online content

Any methods, additional references, Nature Portfolio reporting summaries, source data, extended data, supplementary information, acknowledgements, peer review information; details of author contributions and competing interests; and statements of data and code availability are available at 10.1038/s41591-026-04367-w.

## Supplementary information

Supplementary Information Supplementary Fig. 1: Malaria incidence 2022–2024 by ACD | by year. Supplementary Fig. 2: Malaria incidence 2022–2024 by PCD | Kaplan–Meier survival analysis. Supplementary Fig. 3: Malaria incidence 2022–2024 by PCD | by year. Supplementary Fig. 4: Diarrhea incidence 2022–2024 by ACD | by year. Supplementary Fig. 5: Diarrhea incidence 2022–2024 by PCD | Kaplan−Meier survial analysis. Supplementary Fig. 6: Diarrhea incidence 2022–2024 by PCD | by year. Supplementary Fig. 7: ARIs 2022–2024 by ACD | by year. Supplementary Fig. 8: ARIs 2022–2024 by PCD | Kaplan−Meier survival analysis. Supplementary Fig. 9: ARIs 2022–2024 by PCD | by year. Supplementary Fig. 10: Reduction in costs, embodied carbon, mass and concrete volume. Supplementary Table 1: Key parameters for a Star Home. Supplementary Table 2: Key parameters for a cement block house. Economic assessment. Supplementary Table 3: Sustainable Development Goals addressed by Star Homes.

Reporting Summary

## Figures and Tables

**Extended Data Table 1:**
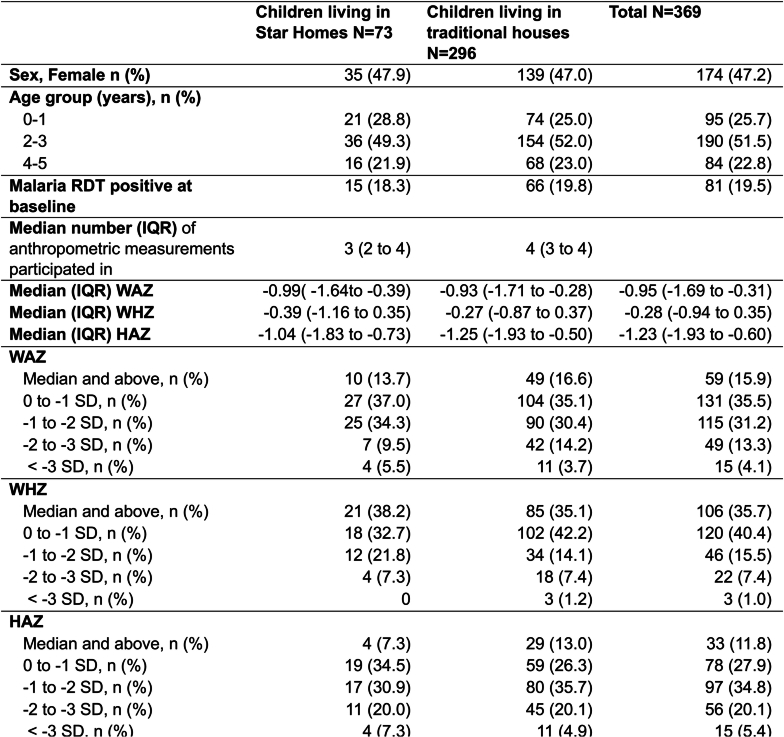
Anthropometry, baseline characteristics Baseline characteristics of the participants, by type of house

**Extended Data Table 2:**
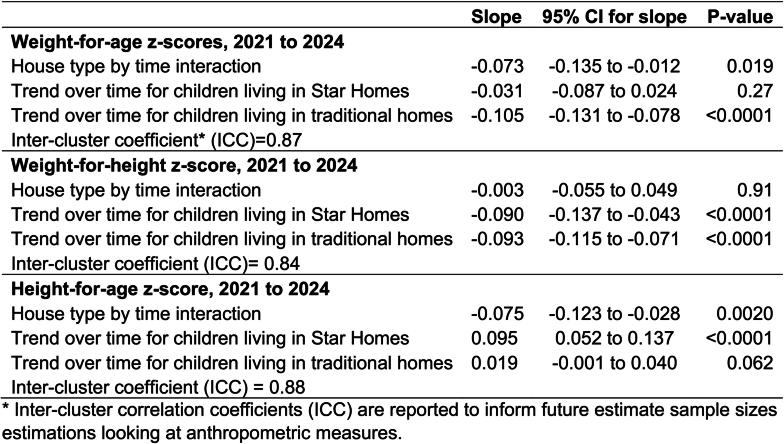
Anthropometry, results Mixed effect model for changes over time of the z-scores, accounting for clustering

**Extended Data Table 3:**
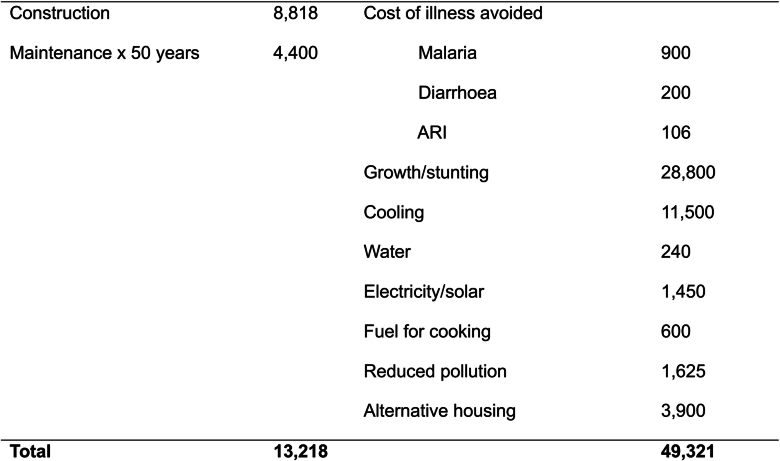
Return on Investment A preliminary order of magnitude estimate of the rate of return of capital invested in a Star Home, evaluated over a 50-year time horizon, accounting for health benefits, lifecycle cost savings, energy efficiency gains, and costs avoided. For more detailed explanations of the estimates please see supplementary materials ‘economic assessment

## Data Availability

Deidentified individual patient data can be requested and may be shared according to the terms defined in the MORU data-sharing policy with other researchers to use in the future from the date of publication. All direct personal identifiers are removed prior to data sharing, and datasets are provided in deidentified form to protect participant confidentiality. All data requests are reviewed by the MORU Data Access Committee in accordance with the MORU Tropical Network Policy on Sharing Data and Other Outputs. Access may require completion of a data access agreement and approval by relevant investigators and ethics committees. Further information on how to apply is on the MORU Tropical Health Network website (https://www.tropmedres.ac/units/moru-bangkok/bioethics-engagement/data-sharing/moru-tropical-network-policy-on-sharing-data-and-other-outputs) or from the corresponding author (lorenz@tropmedres.ac). Decisions regarding approval or rejection of requests will normally be communicated within 4–6 weeks. For approved requests, data access will be granted once the necessary data-sharing agreements and any required ethical approvals have been completed.
