# Supplementary material for: A sustainable house design to improve child health in rural Africa: a cluster-randomized controlled trial
Source: Nat Med. 2026 Apr 21;32(6):2294–301. doi: 10.1038/s41591-026-04367-w (PMC13278959; doi:10.1038/s41591-026-04367-w)
Supplement: Supplementary file 1 — Supplementary Fig. 1: Malaria incidence 2022–2024 by ACD | by year. Supplementary Fig. 2: Malaria incidence 2022–2024 by PCD | Kaplan–Meier survival analysis. Supplementary Fig. 3: Malaria incidence 2022–2024 by PCD | by year. Supplementary Fig. 4: Diarrhea incidence 2022–2024 by ACD | by year. Supplementary Fig. 5: Diarrhea incidence 2022–2024 by PCD | Kaplan−Meier survial analysis. Supplementary Fig. 6: Diarrhea incidence 2022–2024 by PCD | by year. Supplementary Fig. 7: ARIs 2022–2024 by ACD | by year. Supplementary Fig. 8: ARIs 2022–2024 by PCD | Kaplan−Meier survival analysis. Supplementary Fig. 9: ARIs 2022–2024 by PCD | by year. Supplementary Fig. 10: Reduction in costs, embodied carbon, mass and concrete volume. Supplementary Table 1: Key parameters for a Star Home. Supplementary Table 2: Key parameters for a cement block house. Economic assessment. Supplementary Table 3: Sustainable Development Goals addressed by Star Homes. [file 41591_2026_4367_MOESM1_ESM.pdf]

nature medicine

Supplementary information https://doi.org/10.1038/s41591-026-04367-w

A sustainable house design to improve child health in rural Africa: a cluster-randomized controlled trial

In the format provided by the authors and unedited

Supplementary Materials

A sustainable house design to improve child health in rural Africa: a cluster-randomized controlled trial

Table of Contents

Results 2

Figure S1: Malaria incidence 2022 – 24 by ACD | By year 2

Figure S2: Malaria incidence 2022 – 24 by PCD, Kaplan Meyer survival analysis 3

Figure S3: Malaria incidence 2022 – 2024 by PCD | By year 4

Figure S4: Diarrhoea incidence 2022 – 2024 by ACD | By year | 2022 – 2024 5

Figure S5: Diarrhoea incidence 2022 – 2024 by PCD | Kaplan Meyer survial analysis 6

Figure S6: Diarrhoea incidence 2022 – 2024 by PCD | By year 7

Figure S7: Acute Respiratory Tract Infections 2022 – 2024 by ACD | By year 8

Figure S8: Acute Respiratory Tract Infections 2022 – 2024 by PCD | Kaplan Meyer survival analysis 9

Figure S9: Acute Respiratory Tract Infections 2022 – 2024 by PCD | By year 10

Figure S10: Reduction in costs, embodied carbon, mass, and concrete volume 11

Table S1: Key parameters for a Star Home 12

Table S2: Key parameters for a cement block house 13

Economic assessment 14

Table S3: Sustainable Development Goals addressed by Star Homes 18

Results

Figure S1: Malaria incidence 2022 – 24 by ACD | By year

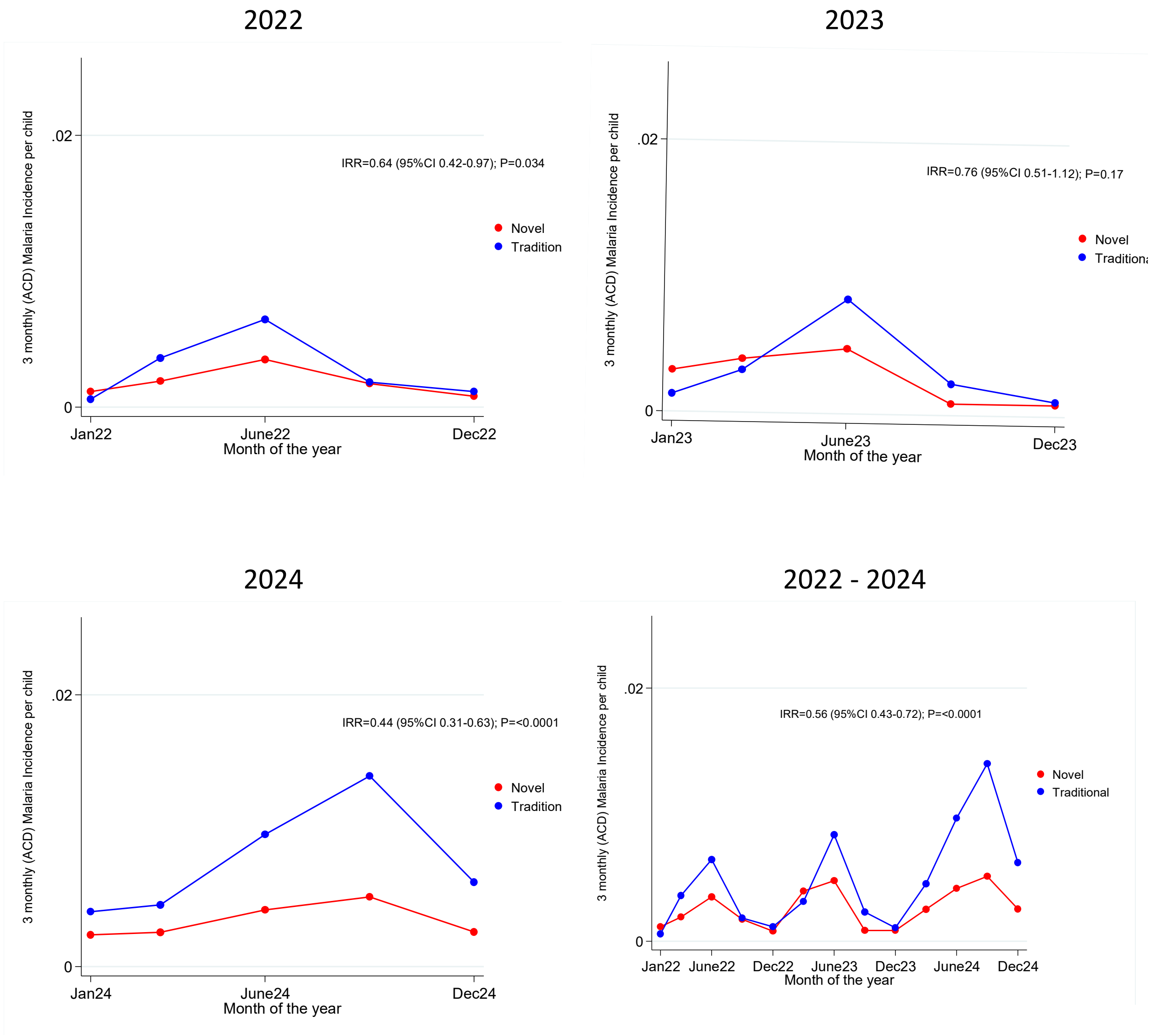

Figure S2: Malaria incidence 2022 – 24 by PCD, Kaplan Meyer survival analysis

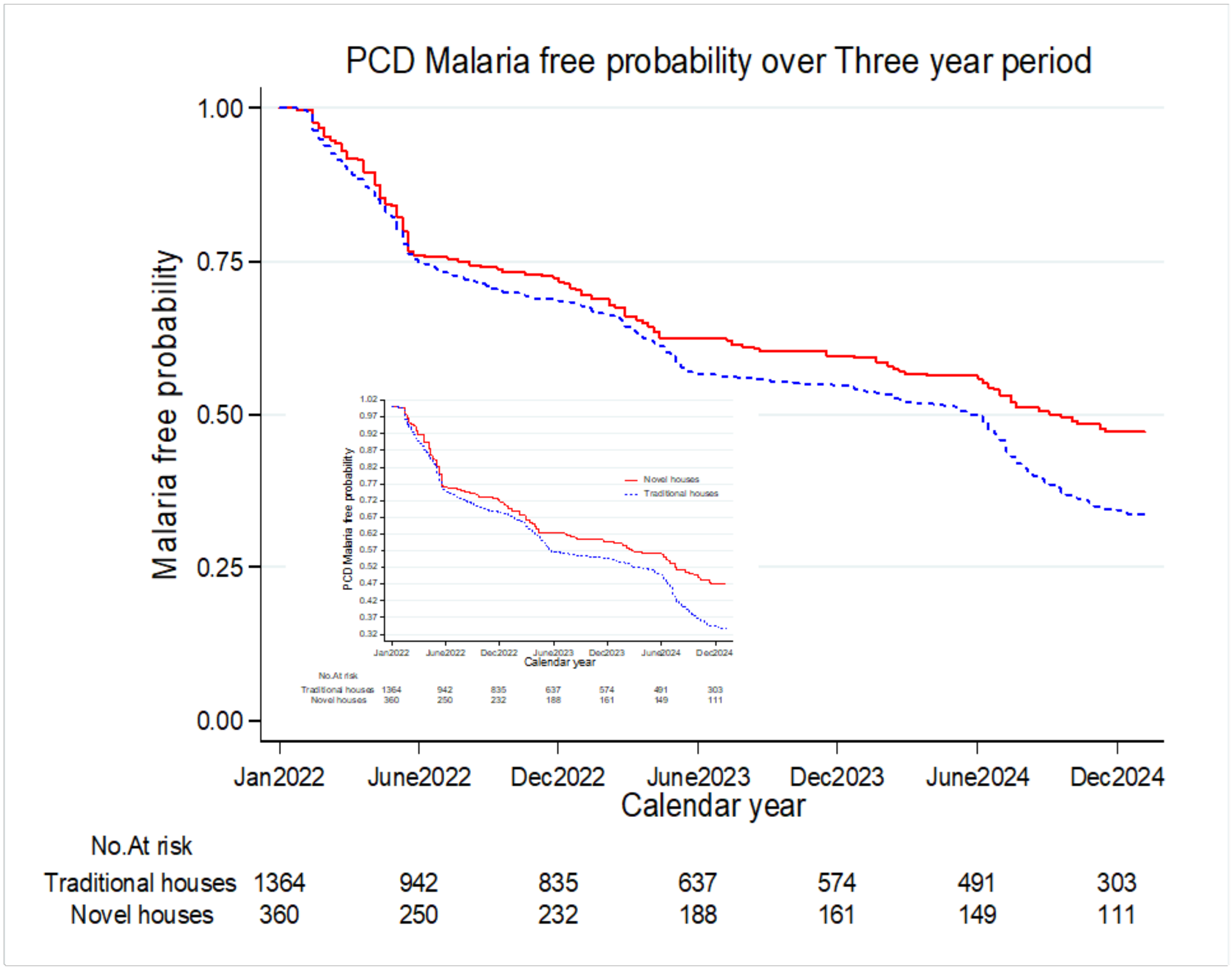

Figure S3: Malaria incidence 2022 – 2024 by PCD | By year

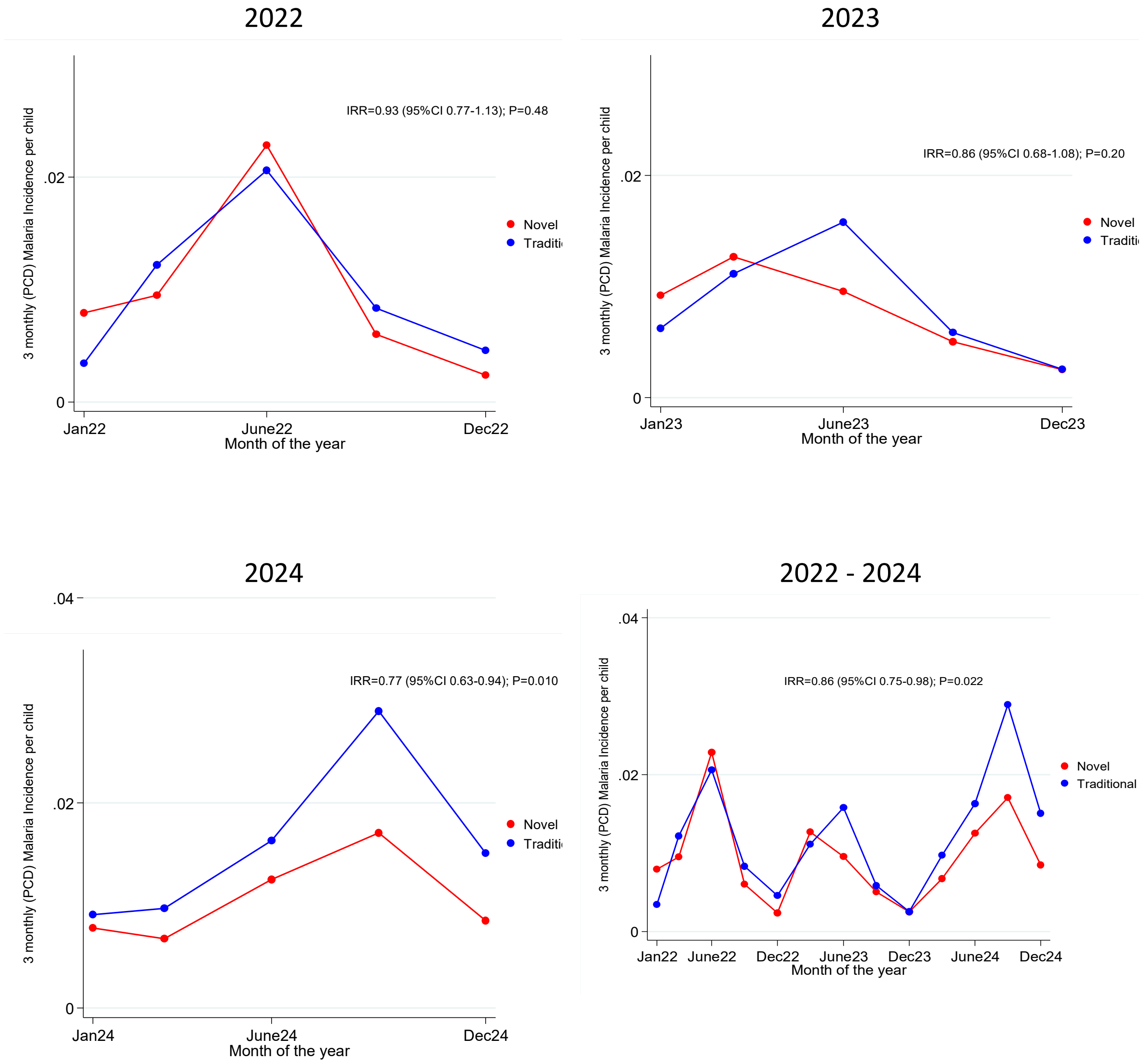

Figure S4: Diarrhoea incidence 2022 – 2024 by ACD | By year | 2022 – 2024

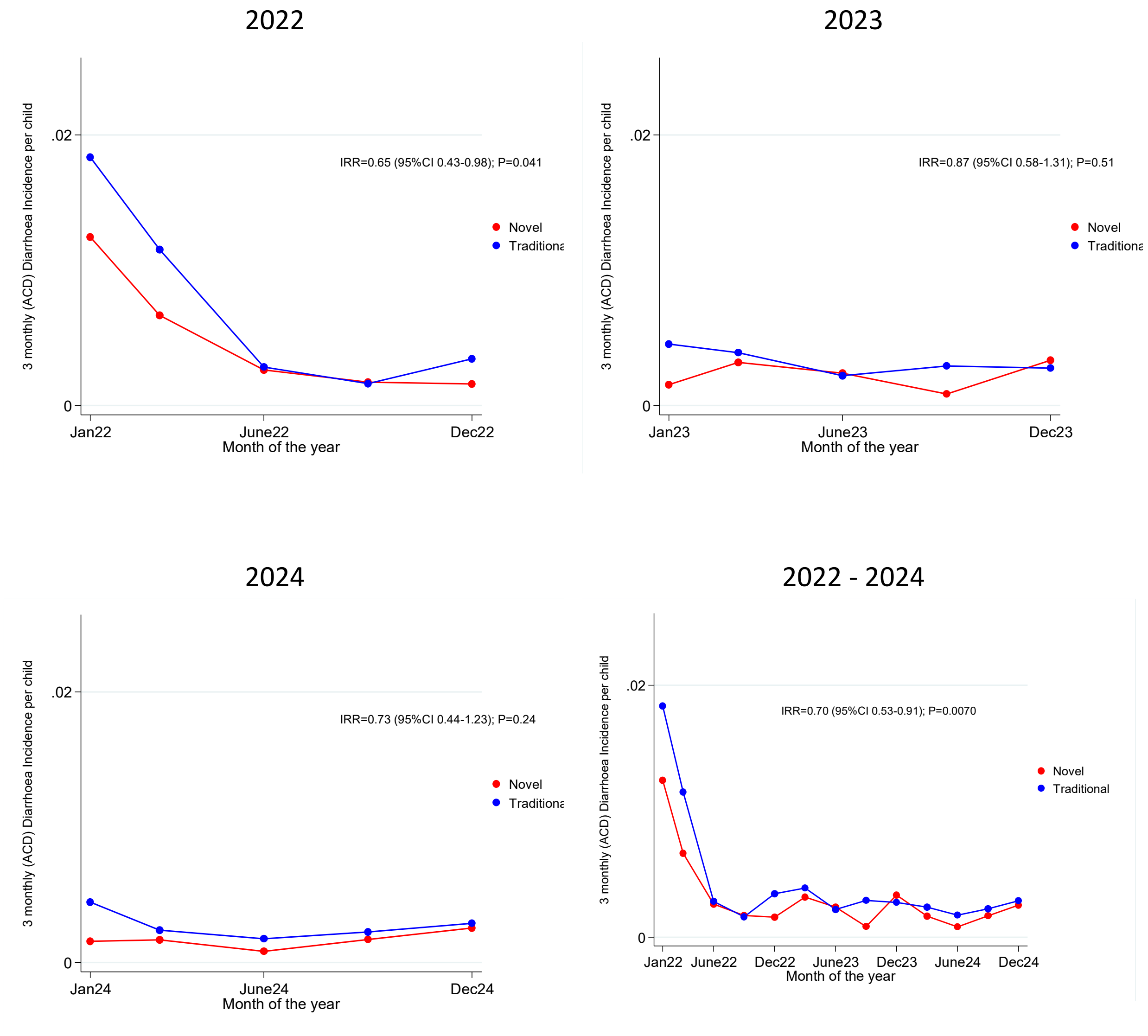

Figure S5: Diarrhoea incidence 2022 – 2024 by PCD | Kaplan Meyer survial analysis

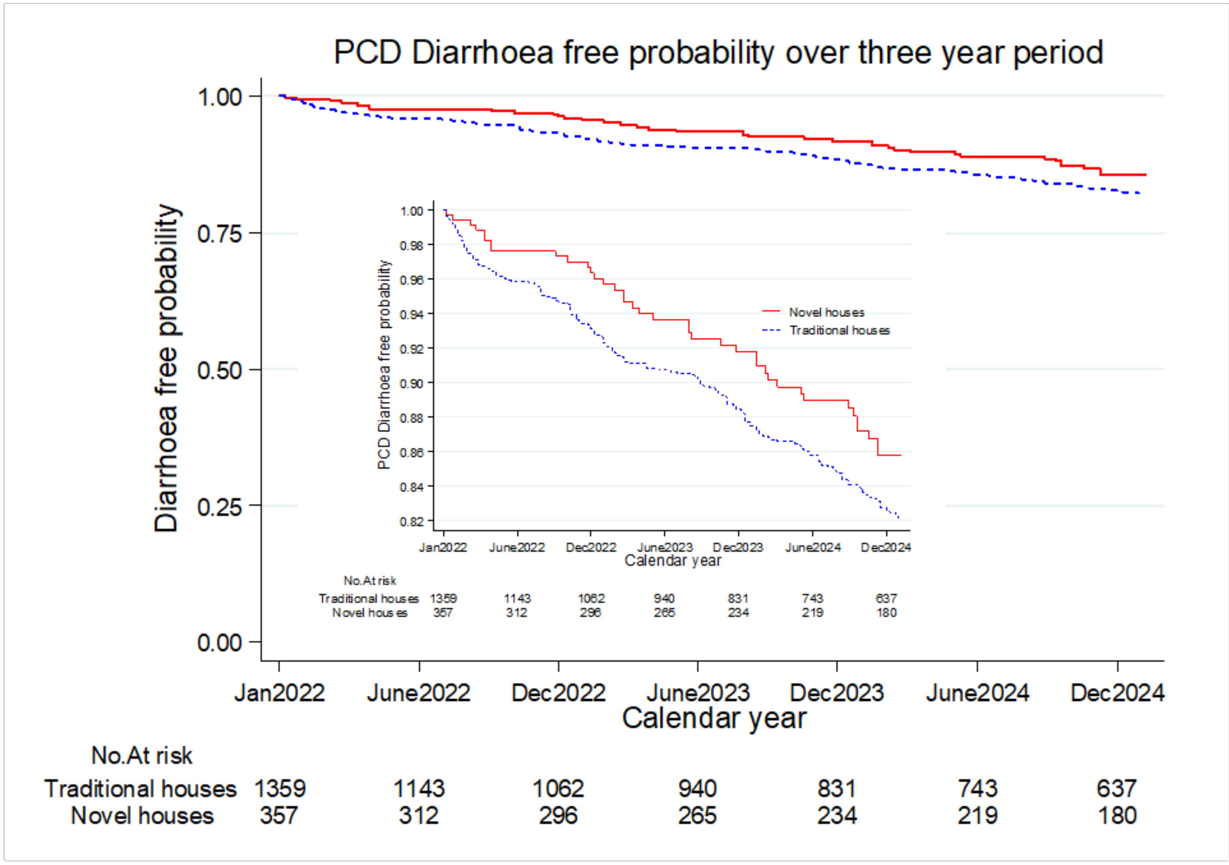

Figure S6: Diarrhoea incidence 2022 – 2024 by PCD | By year

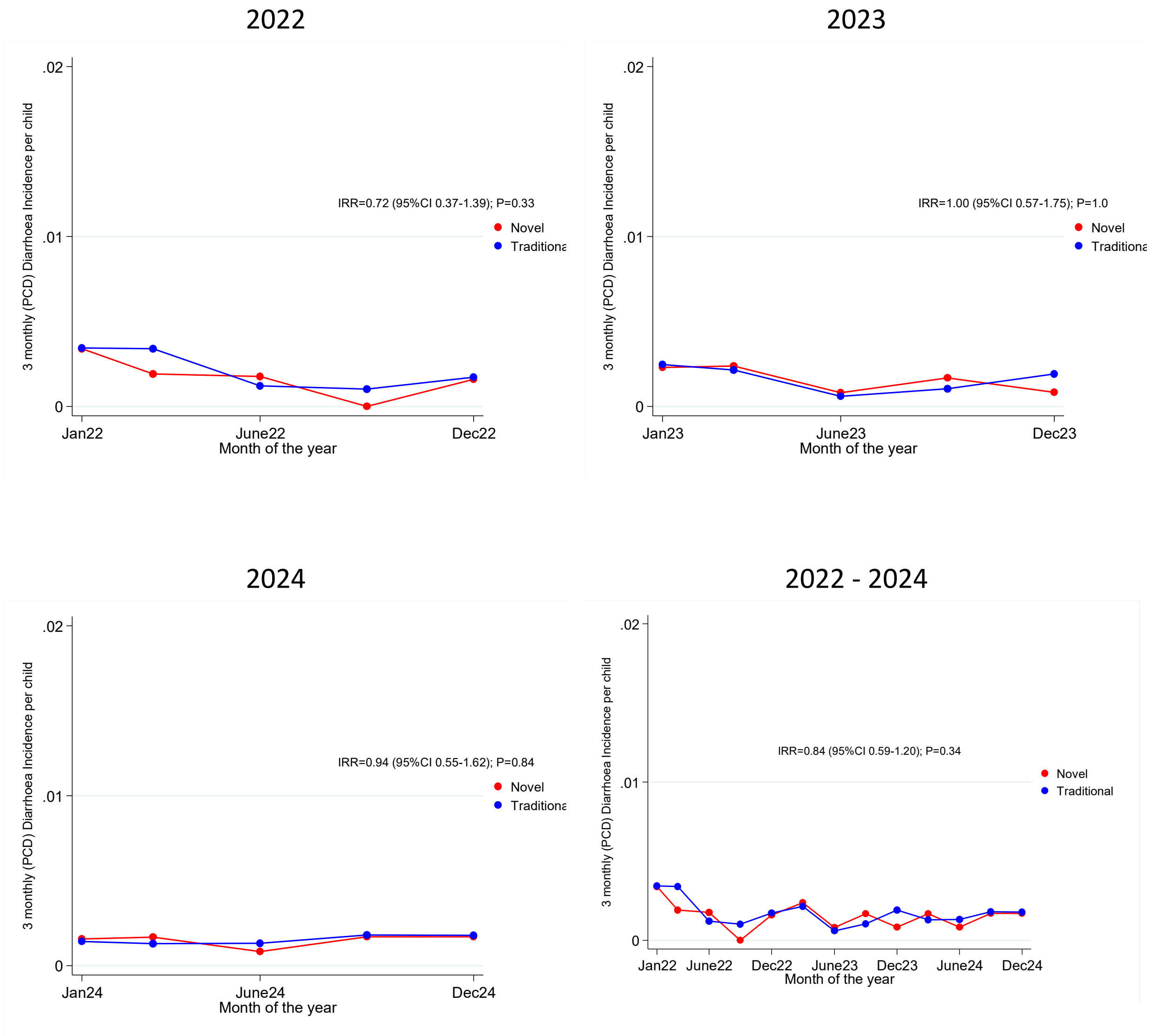

Figure S7: Acute Respiratory Tract Infections 2022 – 2024 by ACD | By year

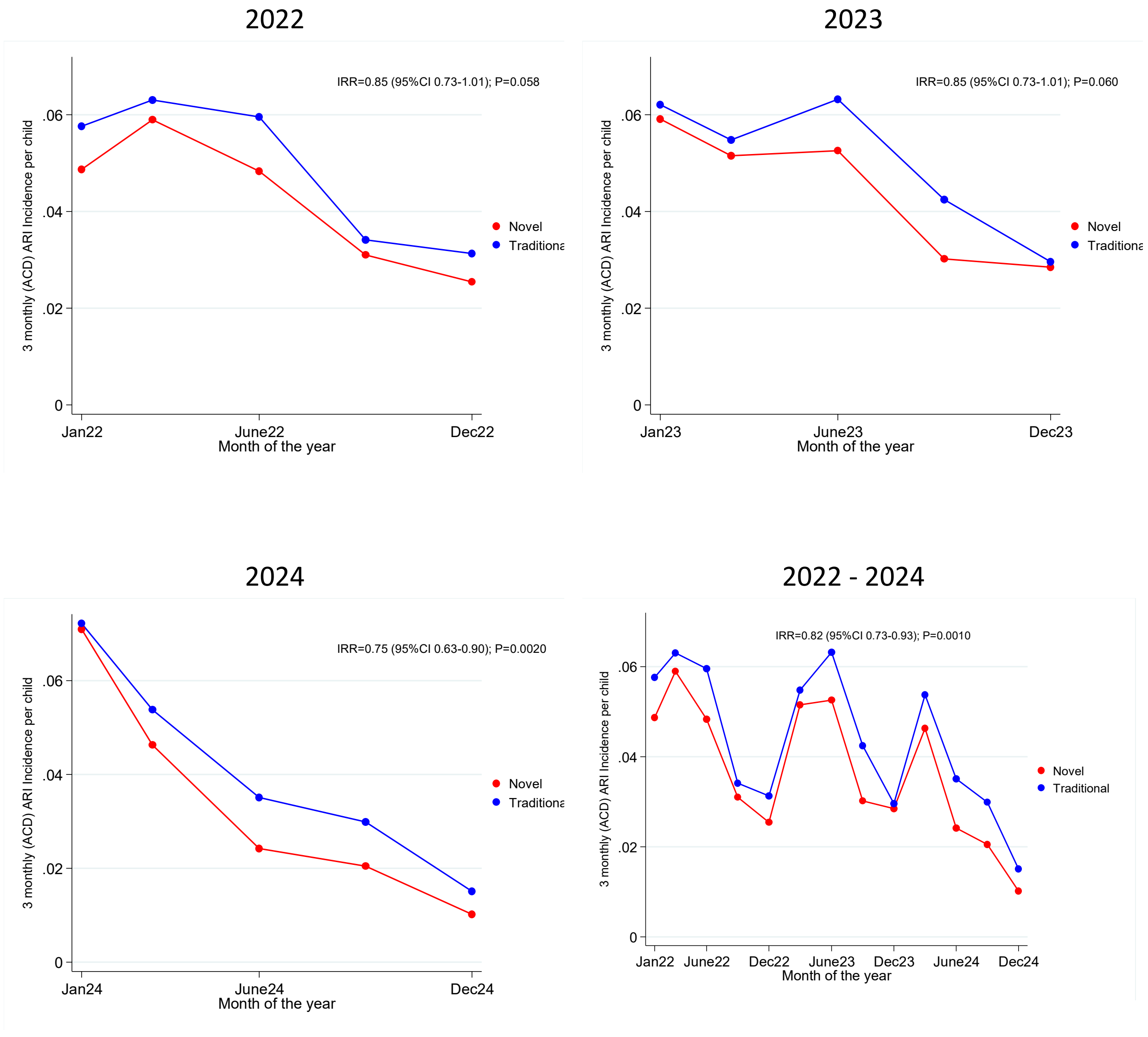

Figure S8: Acute Respiratory Tract Infections 2022 – 2024 by PCD | Kaplan Meyer survival analysis

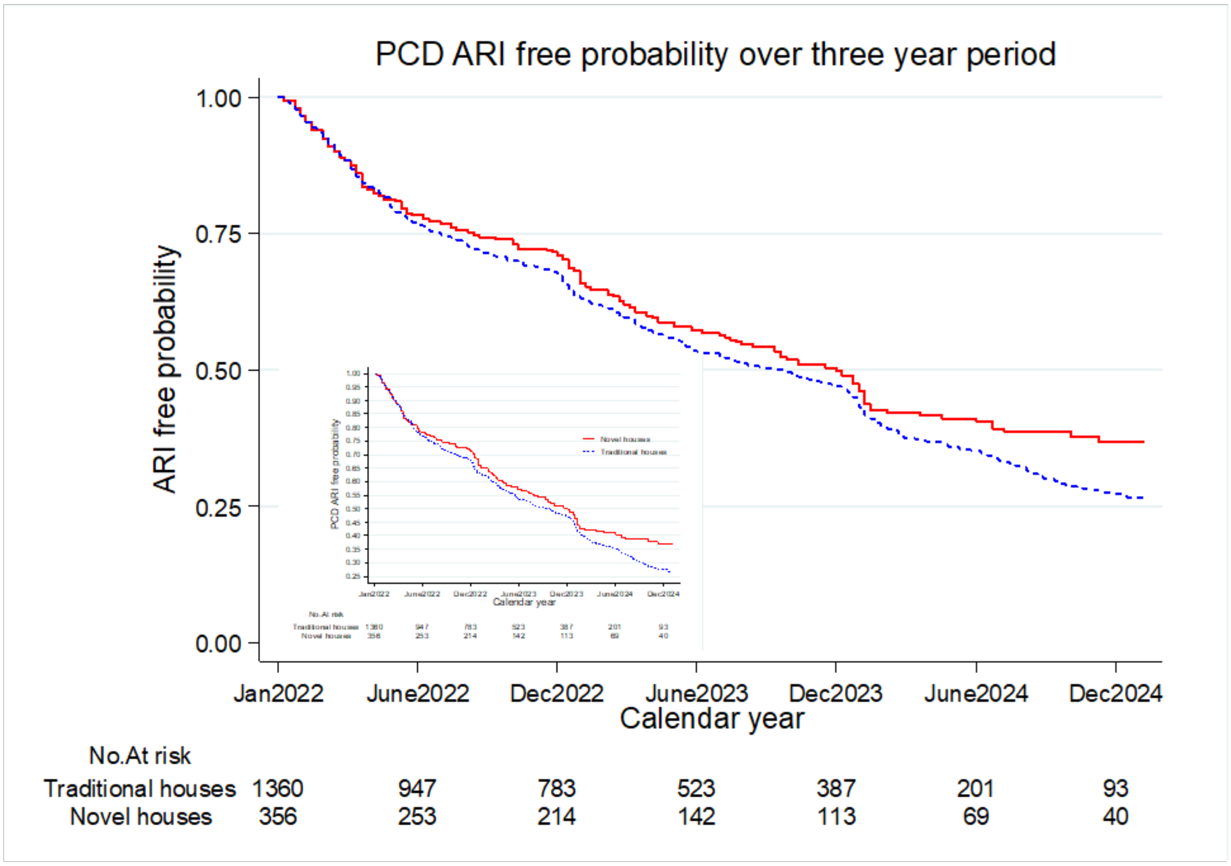

Figure S9: Acute Respiratory Tract Infections 2022 – 2024 by PCD | By year

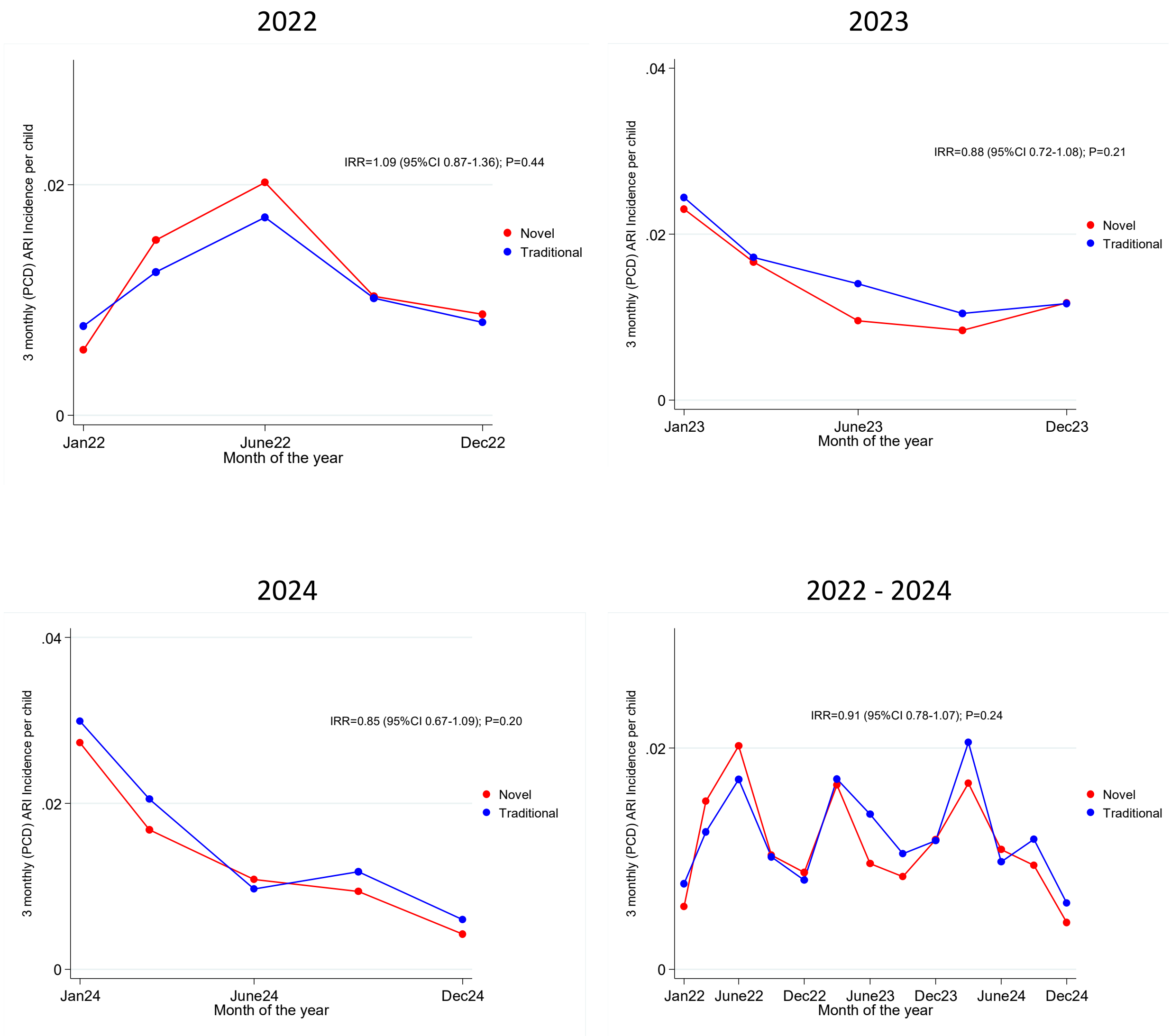

Figure S10: Reduction in costs, embodied carbon, mass, and concrete volume

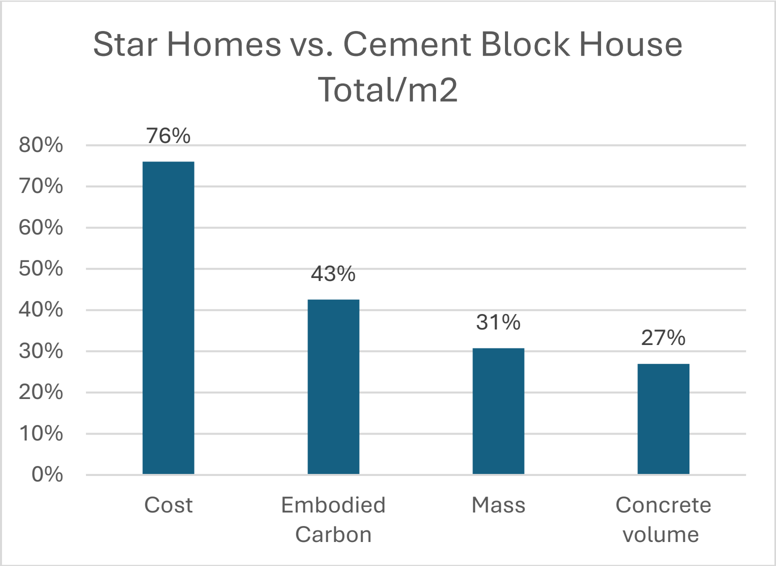

Table S1: Key parameters for a Star Home

Star Home (64,2 m2)	Cost (USD)	Embodied Carbon (kg CO_2)	Mass (kg)	Net Volume (m3)	Density (kg/m3)	Building Material
**Concrete (unreinforced)**						
Concrete walls	15	360	3,271	0.12	2000	Concrete
Stud partition walls	218	400	3,634	1.82	2000	Concrete
Concrete slab first floor	106	194	1,760	0.88	2000	Concrete
**TOTAL**	**338**	**953**	**8,665**	**2.82**		
**Concrete (lightly reinforced)**						
Concrete slab foundation	776	1,448	10,344	4.31	2400	Concrete
**TOTAL**	**776**	**1,448**	**27,673**	**9.95**		
**Aluminum (anodized)**						
Window frames	40	282	33	0.01	2700	Aluminum
Aluminum doors	75	526	61	0.02	2700	Aluminum
**TOTAL**	**115**	**808**	**61**	**0.03**		
**Steel (galvanized)**						
LGS profiles	2,838	245	179	0.02	7850	LGS Steel
Roofing sheets, 28G	805	308	225	0.03	7850	Roofing sheet
Steel mesh in walls	95	114	83	0.11	7850	LGS Steel
Monkey wire mesh	104	125	91	0.12	7850	LGS Steel
**TOTAL**	**3,843**	**792**	**578**	**0.27**		
**Openings**						
Shadenet 100%	79	60	25	0.03	950	Shade Net (HDPE)
Shadenet 80%	121	93	38	0.04	950	Shade Net (HDPE)
Shadenet 40%	37	28	12	0.01	950	Shade Net (HDPE)
**TOTAL**	**237**	**181**	**75**	**0.08**		
**CONCRETE TOTAL**				**12.77**		
**OVERALL TOTAL**	**5,309**	**4,182**	**37,052**			
**TOTAL per m2**	**82.7**	**65.1**	**577**	**0.20**		

Table S2: Key parameters for a cement block house

Cement Block House (64,2 m2)	Cost	Embodied Carbon	Mass	Net Volume	Density	Building Material
	(USD)	(kg CO_2)	(kg)	(m3)	(kg/m3)	
**Concrete (unreinforced)**						
Block - Structural	3,157	5,788	52,618	26.31	2000	Concrete
Hardcore slab	240	115	23,855	10.65	2240	Hardcore (rock)
**TOTAL**	**3,397**	**5,902**	**76,473**	**36.96**		
**Concrete (lightly reinforced)**						
Concrete - Structural	1,876	3,502	25,012	10.42	2400	Concrete
**TOTAL**	**1,876**	**3,502**	**25,012**	**10.42**		
**Wood**						
Window and doors	237	16	317	0.45	700	Hardwood (generalized)
Roof construction	412	31	624	1.25	500	Wood (timber, softwood)
**TOTAL**	**649**	**47**	**624**	**1.70**		
**Steel (galvanized)**						
Roofing sheets, 28G	903	380	277	0.04	7850	Roofing sheet
**TOTAL**	**903**	**380**	**277**	**0.04**		
**Openings**						
Glass	157	89	105	0.04	2500	Glass (float)
**TOTAL**	**157**	**89**	**105**	**0.08**		
**CONCRETE TOTAL**				**47.38**		
**OVERALL TOTAL**	**6,982**	**9,826**	**102,490**			
**TOTAL per m2**	**108.8**	**153.1**	**1,877**	**0.87**		

Economic assessment

Preliminary, order of magnitude estimate of the internal rate of return of capital invested in a Star Home, evaluated over a 50-year time horizon, accounting for health benefits, lifecycle cost savings, energy efficiency gains, and costs avoided.

- **Construction:** The signed contract for 110 The signed contract for 110 star houses constructed by a professional contractor, Eco-homes, Dar es Salaam, was USD 969,980 for 110 houses or USD 8,818 per house including VAT, improved cooking facilities, toilets, gutters, water storage and solar power.
- The estimated **maintenance cost** is based on the 1% rule that 1% of the purchasing price should be annually invested and maintenance. $88 x 50 years= $4,400.

- **Malaria** household costs savings from a 44% reduction in malaria incidence

**Benefit Category**	**Estimated Benefit (USD)**
Health system savings	A study in rural Tanzania estimated the cost of one ACT dose at 0.85 USD. If one dose corresponds per outpatient case, then: 3.38M×0.85≈USD2.9 million3.38M × 0.85 ≈ **USD 2.9 million**<br>This is a lower-bound estimate, excluding diagnostics, facility costs, or hospitalization.
Household out-of-pocket	User experiences suggest malaria treatment at public facilities is mostly free or minimal; private pharmacies may charge up to ~$2 per course. Assuming a modest $2 average out-of-pocket cost per case, the savings would be:<br>3.38M × $2 ≈ **USD 6.8 million**
Productivity gains	Average wage in Tanzania is about 7,310 TZS/hour ≈ $3.13/h (at ~2,337 TZS/USD) (Salary Explorer). Assuming 3 lost workdays per case (8 hours/day):<br>Per case productivity loss ≈ 3 × 8 × $3.13 ≈ $75<br>Applying this to working-age cases (let's say 70% of total):<br>3.38M × 0.70 × $75 ≈ **USD 177 million**
Averted deaths (VSL)	If the case fatality rate is approximately 0.2%:<br>3.38M×0.002=6,760 deaths averted 3.38M × 0.002 = 6,760. Using a conservative Value of Statistical Life (VSL) estimate—say $5,000 per life:<br>6,760 × $5,000 ≈ **USD 33.8 million**
Total	~220 million USD per year
Tanzania population =60 Mio<br>220/60 =$ 3.6/person/year<br>5 person /home 3.6* 5= $ 18<br>**50 years 18*50 = $900**	

- **Diarrhoea** household cost savings from a 30% reduction in diarrhoea incidence

Benefit Category	Value (USD)
Health system savings	Assume average treatment cost = $3/case<br>4.5 million cases×$3=$13.5 million
Household savings	Includes: Medicines bought privately, Transport, Food, Lost time/caregiver wages<br>Estimated cost per case: $2–5<br>Assume $3/case × 4.5M = $13.5 million/year
Productivity gains	Assume 1–2 lost workdays per adult case or per caregiver (for child cases), Wage: ~$3/day in Tanzania, 1 day × $3 × 4.5M = $13.5 million/year
Value of averted deaths	WHO: diarrhoea causes ~8–10% of under-5 deaths. Tanzania under-5 mortality: ~50,000 per year → ~5,000 from diarrhoea<br>A 30% reduction = ~1,500 deaths averted<br>Value of Statistical Life (VSL): Use conservative estimate: $5,000 per life<br>1,500×$5,000=$7.5 million/year1,500 \times \$5,000 = \$7.5
**Total Annual Benefits ~$48 million**	
**48/60*5*50 = USD 200**	

- **ARI** household cost savings and economic benefits of an 18% reduction in Acute Respiratory Infections (ARI) incidence

Benefit Category	Estimated Value (USD)
Health system savings	Outpatient (mild): ~$2–$5<br>Inpatient (severe pneumonia): $25–$50<br>Assumed average treatment cost of $4/case<br>1.8M × $4 = $7.2 million/year
Household savings	Medicines (antibiotics, cough meds), Transport to health facilities<br>Food and caregiving time, Estimated per case: $2–$5<br>Assume $3/case, 1.8M cases × $3 = $5.4 million/year
Productivity gains	Adults missing work due to ARI, Caregivers of sick children missing work<br>Each ARI episode causes ~1–2 lost workdays, Average wage in Tanzania ≈ $3/day, Assume 1 lost day per case, 1.8M × $3 = $5.4 million/year
Averted deaths (VSL)	ARIs (especially pneumonia) are a leading cause of under-5 deaths, Estimated ~7,000–10,000 ARI deaths/year in Tanzania (mostly children), 18% reduction = 1,260–1,800 deaths averted, use conservative VSL = $5,000 per life saved<br>1,500 deaths × $5,000 = $7.5 million/year
**Total Estimated Benefit ~$25.5 million/year**	
**25.5/60*5*50 = 106.25**	

- **Height for age** Estimating the economic benefits of improved height-for-age (HFA) involves quantifying how changes in a child's linear growth (a proxy for long-term nutritional status and health) lead to economic gains over time. A 1 SD increase in HFA at age 2 is associated with 20–30% higher adult earnings, on average.

  Improved HFA is associated with:
  - Better cognitive development
  - Higher educational attainment
  - Higher adult productivity and earnings
  - Lower healthcare costs
  - Reduced mortality

Benefit Category	Estimate
Lifetime productivity gain	GDP per capita = $1,200/year<br>Working life = 40 years<br>Lifetime earnings = $48,000<br>Productivity gain = 20% × $48,000 = $9,600 per child
**3 children per household $ 28,800**	

- **Cooling:**

A more comfortable home that lowers indoor temperature by 2°C, will bring a range of benefits to the household including improved health, productivity gains, and quality of life. For simplicity's sake we estimate here only the direct cost for electricity to run air conditioning during the night (12 hours) to cool indoor temperature by 2°C.

Parameter	Estimate Value
Electricity price	USD 0.088 per kWh
AC energy use assumption	~0.6 kWh per hour
Daily run time	12 hours
Annual energy consumption	≈ 2,628 kWh
Annual cost/household	≈ USD 230
**x 50 years = USD 11,500**	

- **Water:**

Handpump community provided $4,80/year/household

$4.80 x 50 years = $ 240

- **Electricity costs saved by solar power**

Estimate of the electricity cost for basic lighting (illumination only) in an 80 m^2, 2-bedroom home in East Africa.

For an 80 m^2, 2-bedroom home → 84 kWh × 4 = 336 kWh/year. Price USD/kWh = 0.086 ≈$29/year.

- **Fuel for cooking**

Cost savings from using an improved cookstove (ICS) for a household of five people in East Africa, based on fuel, financial, and time efficiencies. A traditional 3 stone open fire consumes gathered wood: ~$52 per year. ICS using gathered wood: ~$40 per year.

→ **Savings: $12 annually per household/year. X 50 years = $600**

- **Pollution**

Estimating household-level cost savings from a 20–30% reduction in air pollution—and thereby lower non-communicable disease (NCD) burden—in East Africa is complex. Here an illustrative approximation based on regional and global data.

- Reduced healthcare expenses (doctor visits, medication, hospital stays),
- Improved productivity (fewer sick days, better work/school attendance),
- Enhanced cognitive outcomes (especially for children),
- Plus environmental and social gains.

A 20–30% reduction in air pollution would likely translate into per person savings of $6.50–$9.75/year. Using the minimum estimate of $6.5/person for a 5 person household over 50 years = $1,625

- **Alternative accommodation** (wattle and daub construction i.e. mud hut)

For a local farmer in rural Tanzania, constructing a basic mud hut typically costs between **USD 50 and USD 400**, depending on size and materials, with USD 390 being a reasonable average for durable family-built homes using local resources. The mud hut needs to be repaired after each rain and is completely replaced after maximally 5 years.

Average traditional hut (family-built)	~USD 390
More elaborate or larger structure	Up to USD 1,000

Over a 50 year period 10 huts at $390 are constructed= $3,900

Table S3: Sustainable Development Goals addressed by Star Homes

- **SDG #3 Good health and Wellbeing**
    - Target 3.3. end malaria
    - Target 3.1 end preventable deaths under 5
- **SDG #6 Clean water and sanitation**
    - Target 6.1 access to safe and affordable drinking water
    - Target 6.2 adequate and equitable sanitation and hygiene for all and end open defecation
- **SDG #7 Affordable and clean energy**
    - Target 7.1 domestic resource mobilization – building materials, solar energy, smoke free stoves
- **SDG #11 Sustainable cities and communities**
    - Target 11.1 access for all to adequate, safe and affordable housing
- **SDG #13 Climate action**
    - Target 13.b climate change-related planning and management in least developed countries
        - Response to increasing temperatures and extreme climate events
            - Passive cooling through cross-ventilation, nighttime cooling
        - Prevention of global warming through economic building
            - Star Homes less embodied carbon, less concrete

***
