# Supplementary material for: A sustainable house design to improve child health in rural Africa: a cluster-randomized controlled trial
Source: Nat Med. 2026 Apr 21;32(6):2294–301. doi: 10.1038/s41591-026-04367-w (PMC13278959; doi:10.1038/s41591-026-04367-w)
Supplement: Supplementary file 2 — Reporting Summary [file 41591_2026_4367_MOESM2_ESM.pdf]

nature portfolio

Corresponding author(s): Lorenz von Seidlein

Last updated by author(s): Mar 16, 2026

Reporting Summary

Nature Portfolio wishes to improve the reproducibility of the work that we publish. This form provides structure for consistency and transparency in reporting. For further information on Nature Portfolio policies, see our Editorial Policies and the Editorial Policy Checklist.

Statistics

For all statistical analyses, confirm that the following items are present in the figure legend, table legend, main text, or Methods section.

n/a	Confirmed	
☐	☒	The exact sample size (*n*) for each experimental group/condition, given as a discrete number and unit of measurement
☒	☐	A statement on whether measurements were taken from distinct samples or whether the same sample was measured repeatedly
☐	☒	The statistical test(s) used AND whether they are one- or two-sided<br>*Only common tests should be described solely by name; describe more complex techniques in the Methods section.*
☐	☒	A description of all covariates tested
☐	☒	A description of any assumptions or corrections, such as tests of normality and adjustment for multiple comparisons
☐	☒	A full description of the statistical parameters including central tendency (e.g. means) or other basic estimates (e.g. regression coefficient) AND variation (e.g. standard deviation) or associated estimates of uncertainty (e.g. confidence intervals)
☐	☒	For null hypothesis testing, the test statistic (e.g. *F*, *t*, *r*) with confidence intervals, effect sizes, degrees of freedom and *P* value noted<br>*Give P values as exact values whenever suitable.*
☒	☐	For Bayesian analysis, information on the choice of priors and Markov chain Monte Carlo settings
☐	☒	For hierarchical and complex designs, identification of the appropriate level for tests and full reporting of outcomes
☒	☐	Estimates of effect sizes (e.g. Cohen's *d*, Pearson's *r*), indicating how they were calculated

Our web collection on statistics for biologists contains articles on many of the points above.

Software and code

Policy information about availability of computer code

Data collection	Field data were collected on tablets.in a custom made "bespoke" software.
Data analysis	The prespecified analysis was performed in Stata software (Version 18, College Station, TX, USA).

For manuscripts utilizing custom algorithms or software that are central to the research but not yet described in published literature, software must be made available to editors and reviewers. We strongly encourage code deposition in a community repository (e.g. GitHub). See the Nature Portfolio guidelines for submitting code & software for further information.

Data

Policy information about availability of data

All manuscripts must include a data availability statement. This statement should provide the following information, where applicable:

- Accession codes, unique identifiers, or web links for publicly available datasets
- A description of any restrictions on data availability
- For clinical datasets or third party data, please ensure that the statement adheres to our policy

De-identified Iindividual patient data can be requested and may be shared according to the terms defined in the Mahidol Oxford Tropical Medicine Research Unit data sharing policy with other researchers to use in the future from the date of publication. All direct personal identifiers are removed prior to data sharing, and datasets are provided in de-identified form to protect participant confidentiality. All data requests are reviewed by the MORU Data Access Committee in accordance with the MORU Tropical Network Policy on Sharing Data and Other Outputs. Access may require completion of a data access agreement and approval by relevant

investigators and ethics committees. Further information on how to apply is on the Mahidol Oxford Tropical Medicine Research Unit Tropical Health Network website https://www.tropmedres.ac/units/moru-bangkok/bioethics-engagement/data-sharing/moru-tropical-network-policy-on-sharing-data-and-other-outputs or from the corresponding author (lorenz@tropmedres.ac). Decisions regarding approval or rejection of requests will normally be communicated within 4–6 weeks. For approved requests, data access will be granted once the necessary data-sharing agreements and any required ethical approvals have been completed.

Research involving human participants, their data, or biological material

Policy information about studies with human participants or human data. See also policy information about sex, gender (identity/presentation), and sexual orientation and race, ethnicity and racism.

Reporting on sex and gender	An exploratory Multivariable analysis for the primary outcome did not find a differential impact by sex/gender. Furthermore, univariate and multivariable (adjusted) analyses (that included gender/sex) for the primary outcome produced exactly the same results for the intervention effect. This confirms that there is no evidence of gender differences in terms of intervention effect (i.e. gender/sex does not confound the outcome from the intervention, house type). In the study design, we allowed all participants to be included in the study without preference for any sex/gender; and both sexes/all genders were treated equally in the design, study conduct, reporting and analysis.
Reporting on race, ethnicity, or other socially relevant groupings	We report the study site but not the ethnic groups participating in the trial..<br>The population of the Mtwara region in southern Tanzania is predominantly composed of three major ethnic groups: the Makonde, the Yao, and the Makua. Within the study villages involved in the Star Homes project, the Makonde constitute the dominant group. The Makonde differ in various cultural aspects, including belief systems, social organization, and customary practices, from other ethnic groups in Tanzania. Like many coastal communities along East Africa, the Makonde are primarily Muslim, although a minority identify as Christian or adhere to traditional religious systems involving animistic forms of ancestor veneration. Historically, the Makonde are a matrilineal society, meaning that lineage, inheritance, and social affiliation are traced through the mother. As part of this system, husbands traditionally relocate to the villages of their wives after marriage. The primary language spoken is ChiMakonde (or Makonde), although Kiswahili (or Swahili), Tanzania's national and official language, is also widely spoken.
Population characteristics	Baseline characteristics of the participants by type of house design are shown in Table 1.
Recruitment	Mtwara district was selected as study site based on a) high malaria prevalence b) hot, humid climate, c) accessibility and d) relatively limited vector-control interventions and medical research activities compared with northern Tanzania (Extended Data 1)13. Villages were eligible for inclusion if they had: 1) a population exceeding 100 households, 2) road accessibility throughout the year, 3) no connection to the national electricity grid, 4) no municipal water supply, and 5) high malaria prevalence. Ultimately 59 villages were found eligible and lotteries for a new home were conducted among households that had at least two children under 13 years of age, were living in a mud hut (wattle-and-daub walls, thatched roof, and an earth floor, had a temporary or no latrine, had access to sufficient land for construction, and were willing to participate in a three-year follow-up period
Ethics oversight	The trial protocol was approved by the Tanzanian National Institute for Medical Research (NIMR/HQ/R.8a/Vol.IX/3695) and the Oxford Tropical Research Ethics Committee (OxTREC 533-20).

Note that full information on the approval of the study protocol must also be provided in the manuscript.

Field-specific reporting

Please select the one below that is the best fit for your research. If you are not sure, read the appropriate sections before making your selection.

☒ Life sciences ☐ Behavioural & social sciences ☐ Ecological, evolutionary & environmental sciences

For a reference copy of the document with all sections, see nature.com/documents/nr-reporting-summary-flat.pdf

Life sciences study design

All studies must disclose on these points even when the disclosure is negative.

Sample size	For the purpose of estimating sample size, we made the assumption of 10 malaria episodes per 100 children per year in the control households, with a projected 30% decrease to seven malaria episodes per 100 children per year in the Star Homes11. To detect this difference with 80% statistical power and a 5% significance level, we determined that 105 households with three child-years of observation each would be required per group. Assuming that malaria was likely to have a lower incidence than diarrhoeal diseases and ARIs, the sample size calculated for malaria should be adequate for detecting significant differences in the incidence of all three diseases. To account for potential loss to follow-up, we enrolled 110 households into the intervention group. Given the necessity to adjust for clustering by both village and household during analysis, power simulations were conducted using a Poisson random effects model to determine the optimal ratio of traditional to Star Home households that would ensure adequate statistical power, assuming a coefficient of variation (CV) of 0.2512. For every intervention household, a minimum of four control households was deemed necessary. Due to the limited information available in the existing literature regarding intra-cluster correlations (ICCs) at the village and household levels, we opted for a conservative estimate of a baseline ICC of 0.613.
Data exclusions	No data were excluded
Replication	This study was a prospective field trial conducted over three years. Because of the scale and context of the intervention, direct replication of the trial under identical conditions is not feasible. However, all primary and secondary outcomes were pre-specified in the study protocol, and

standardized procedures were used for participant recruitment, surveillance, data collection, and laboratory diagnostics. Statistical analyses were conducted using pre-specified models and were independently verified using the study dataset. De-identified individual participant data and analysis code can be made available upon reasonable request through the MORU Tropical Health Network Data Access Committee, allowing independent researchers to reproduce the reported analyses.

Randomization

Participants were randomly assigned to study groups using a lottery-based allocation procedureThe lottery was conducted over two rounds. In each village, the names of eligible and interested heads of households were inscribed written on slips of paper, each placed in an individual envelope. All envelopes were put in a transparent bucket and mixed by a village volunteer schoolchild, who in public drew between one and three envelopes depending on the village's size. The winners became owners of Star Homes. The remaining envelopes were included in the next round to select 4 to 12 additional envelopes. The winners of the second round were invited to serve as comparison households. A dynamic cohort of children residing in 110 Star Homes (intervention) and 513 traditional African households (control) was recruited.

Blinding

The intervention, the house, could not be blinded. Laboratory procedures (PCR) were single blinded.

Reporting for specific materials, systems and methods

We require information from authors about some types of materials, experimental systems and methods used in many studies. Here, indicate whether each material, system or method listed is relevant to your study. If you are not sure if a list item applies to your research, read the appropriate section before selecting a response.

Materials & experimental systems

n/a	Involved in the study	
☒	☐	Antibodies
☒	☐	Eukaryotic cell lines
☒	☐	Palaeontology and archaeology
☒	☐	Animals and other organisms
☐	☒	Clinical data
☒	☐	Dual use research of concern
☒	☐	Plants

Methods

n/a	Involved in the study	
☒	☐	ChIP-seq
☒	☐	Flow cytometry
☒	☐	MRI-based neuroimaging

Clinical data

Policy information about clinical studies

All manuscripts should comply with the ICMJE guidelines for publication of clinical research and a completed CONSORT checklist must be included with all submissions.

Clinical trial registration

ClinicalTrials.gov NCT04 529434

Study protocol

has been published and submitted with the manuscript.

Data collection

Data were collected in rual villages in Mtwara,Tanzania. Data collection started in 4th January 2021 and ended in 31st December 2024.

Outcomes

The primary outcome was the incidence of Plasmodium falciparum malaria detected through active case detection (ACD) during weekly household visits. Secondary clinical outcomes included the incidence of P. falciparum malaria detected through passive case detection (PCD), as well as the incidence of childhood diarrhoeal disease and acute respiratory infections (ARI), measured through both ACD and PCD. Incidence rates were expressed as the number of episodes per 1,000 person-years of observation, and differences between study groups were estimated using incidence rate ratios (IRR) adjusted for intervention status, age, sex, and season. Parasitological outcomes included P. falciparum prevalence measured during cross-sectional malaria surveys using molecular diagnostics. Child growth and nutritional status were assessed in a nested anthropometric cohort of children under five years of age. Anthropometric outcomes included height-for-age, weight-for-age, and weight-for-height z-scores. Economic outcomes included an assessment of the cost and carbon footprint of Star Homes compared with conventional rural housing, as well as an exploratory cost–benefit analysis estimating the long-term return on investment associated with the intervention.

Plants

Seed stocks	*Report on the source of all seed stocks or other plant material used. If applicable, state the seed stock centre and catalogue number. If plant specimens were collected from the field, describe the collection location, date and sampling procedures.*
Novel plant genotypes	*Describe the methods by which all novel plant genotypes were produced. This includes those generated by transgenic approaches, gene editing, chemical/radiation-based mutagenesis and hybridization. For transgenic lines, describe the transformation method, the number of independent lines analyzed and the generation upon which experiments were performed. For gene-edited lines, describe the editor used, the endogenous sequence targeted for editing, the targeting guide RNA sequence (if applicable) and how the editor was applied.*
Authentication	*Describe any authentication procedures for each seed stock used or novel genotype generated. Describe any experiments used to assess the effect of a mutation and, where applicable, how potential secondary effects (e.g. second site T-DNA insertions, mosiacism, off-target gene editing) were examined.*
